# Computation of Electrical Conductivities of Aqueous Electrolyte Solutions: Two Surfaces, One Property

**DOI:** 10.1021/acs.jctc.3c00562

**Published:** 2023-07-28

**Authors:** Samuel Blazquez, Jose L. F. Abascal, Jelle Lagerweij, Parsa Habibi, Poulumi Dey, Thijs J. H. Vlugt, Othonas A. Moultos, Carlos Vega

**Affiliations:** † Dpto. Química Física I, Fac. Ciencias Químicas, Universidad Complutense de Madrid, 28040 Madrid, Spain; ‡ Engineering Thermodynamics, Process and Energy Department, Faculty of Mechanical, Maritime and Materials Engineering, Delft University of Technology, Leeghwaterstraat 39, 2628CB Delft, The Netherlands; § Department of Materials Science and Engineering, Faculty of Mechanical, Maritime and Materials Engineering, Delft University of Technology, Mekelweg 2, 2628CD Delft, The Netherlands

## Abstract

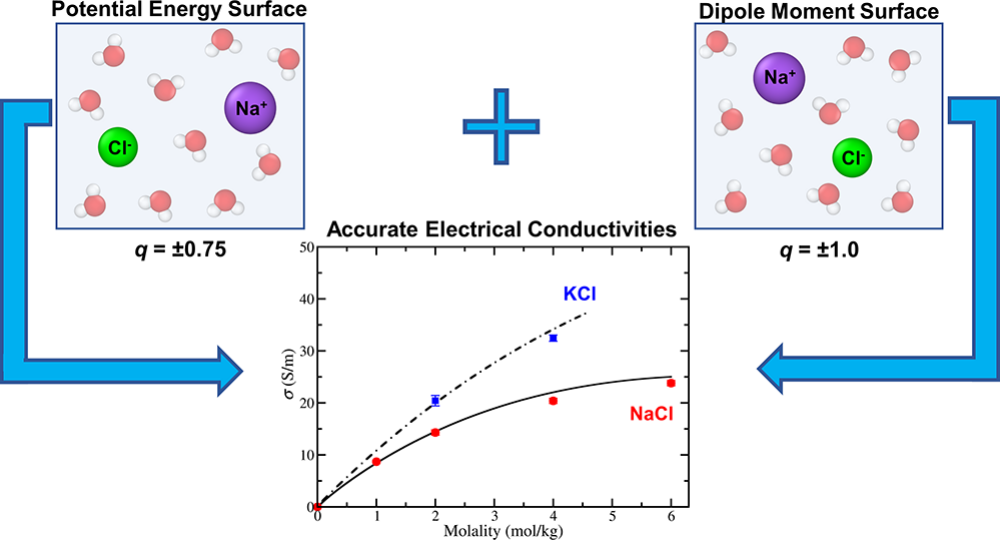

In this work, we computed electrical conductivities under ambient conditions of aqueous NaCl and KCl solutions by using the Einstein–Helfand equation. Common force fields (charge *q* = ±1 *e*) do not reproduce the experimental values of electrical conductivities, viscosities, and diffusion coefficients. Recently, we proposed the idea of using different charges to describe the potential energy surface (PES) and the dipole moment surface (DMS). In this work, we implement this concept. The equilibrium trajectories required to evaluate electrical conductivities (within linear response theory) were obtained by using scaled charges (with the value *q* = ±0.75 *e*) to describe the PES. The potential parameters were those of the Madrid-Transport force field, which accurately describe viscosities and diffusion coefficients of these ionic solutions. However, integer charges were used to compute the conductivities (thus describing the DMS). The basic idea is that although the scaled charge describes the ion–water interaction better, the integer charge reflects the value of the charge that is transported due to the electric field. The agreement obtained with experiments is excellent, as for the first time electrical conductivities (and the other transport properties) of NaCl and KCl electrolyte solutions are described with high accuracy for the whole concentration range up to their solubility limit. Finally, we propose an easy way to obtain a rough estimate of the actual electrical conductivity of the potential model under consideration using the approximate Nernst–Einstein equation, which neglects correlations between different ions.

## 1 Introduction

Electrolyte solutions are ubiquitous in nature, where ions can play a key role in many living organisms.^1^ Electrolytes are also important in many other fields such as battery technology^2−4^ and desalination processes.^5^ Electrolyte solutions have always been the subject of scientific interest^1,6−9^ and computer simulations can be a valuable tool for the study of complex phenomena related to these electrolyte solutions in combination with experimental studies. In the 1970s, Heizinger, Vogel, Singer, and Sangster^10−14^ published the first simulation studies of ionic systems. However, the computational cost of the simulations and the lack of suitable force fields for water and electrolytes did not allow major advances until recent years.

Computational simulations are useful tools for studying phenomena elusive to experiments and for predicting properties of interest. Nevertheless, a suitable force field for the studied system is needed. In the case of aqueous electrolyte solutions, force fields for water and ions are necessary.^15^ In the case of water, the first force field was proposed by Bernal and Fowler in 1933.^15^ Fifty years later, Jorgensen and co-workers started to develop new potential models, such as the TIP3P,^16^ the TIP4P,^16^ and the TIP5P^17^ force fields. At the same time, the popular SPC/E force field was developed by Berendsen and co-workers.^18^ Later, in the 2000s, the knowledge gained from the aforementioned models allowed the development of two of the water models that best reproduce a wide range of properties, which are TIP4P-Ew^19^ and TIP4P/2005.^20^ In fact, the TIP4P/2005 potential is able to reproduce a variety of properties such as densities, viscosities, and the temperature of the maximum in density (TMD).^21−23^ There is no classical model able to reproduce all properties of pure water.^21,24^ One option to improve results of the previous mentioned force fields is to use polarizable models, such as the HBP,^25^ the MB-Pol,^26^ or the BK3.^27^ However, these force fields are between three and ten times more computationally expensive than the nonpolarizable force fields and are also not able to reproduce certain properties simultaneously, such as TMD and melting temperature.^24^ Besides water models, ion–ion and ion–water interactions have to be described to study electrolyte solutions. It is in the recent years that a large variety of force fields for salts have been proposed.^28−61^ Among the most popular force fields for ions, we can find the one proposed by Joung and Cheatham^42^ (JC), which includes all the alkali halides. These can be used in combination with three different water models, namely, TIP3P, TIP4P-Ew, and SPC/E with the JC-SPC/E being the one that provides overall better results. The other popular force field that is widely used in the literature is the one developed by Smith and Dang^34^ (SD) in combination with SPC/E water.^18^ Although these force fields have been quite successful in describing many properties of electrolytes (e.g., densities, structure), they fail in describing properties such as solubilities, viscosities and activity coefficients.^62^

In an attempt to overcome the limitations of current force fields for electrolytes, the idea of using scaled charges for the ions was suggested. The concept of scaled charges (i.e., assign a charge smaller than one for monovalent ions) arises from the work of Leontyev and Stuchebrukhov^63−68^ who proposed a charge of ±0.75 (in electron units) for ions in solution (this was also denoted as Electronic Continuum Correction, ECC). The use of scaled charges has also been proposed by Kann and Skinner.^69^ However, in this case, the value of the scaled charge is selected in such a way that the potential of mean force between ions at infinite dilution and large distances should be the same in experiments and in the force field (so that the model recovers the experimental Debye–Huckel law at infinite dilution). As the potential of mean force depends on the dielectric constant of the water model, so does the value of the scaled charge, leading to a value of ±0.85 in the particular case of water when described by the TIP4P/2005 model. The use of scaled charges for ions in solution has undergone a significant expansion in the last years. Different groups have proposed new force fields with scaled charges, including those of Jungwirth and co-workers,^70−74^ Barbosa and co-workers,^75,76^ Li and Wang,^77^ and Bruce and van der Vegt.^78^ Other authors have proposed the use of different scaled charges for the cation and anion (charging the surrounding water molecules to maintain the electroneutrality of the system).^79−83^ Breton and Joly also studied the effect of introducing scaled charges for studying interfacial properties.^84^ In this context, we developed a model for NaCl based on scaled charges.^48^ Later, we considered a larger number of salts and we proposed the Madrid-2019 force field,^58,59^ which includes all the possible alkali halides, some divalent salts (Mg^2+^ and Ca^2+^), and sulfates (SO_4_^2–^). We have shown in previous works that this force field is able to reproduce different properties of interest, such as the salting out effect of methane,^85^ the TMD of different salt solutions,^86^ the freezing depression of ice in the presence of different electrolytes,^87^ or different properties of seawater.^88^ Although scaled charges improve the results in the majority of properties with respect to unit charge models, there is no unique value of the scaled charge that describes all properties correctly. As we have recently shown, the scaled charge can be taken as a fitting parameter depending on the property that one wants to reproduce.^61^ Transport properties are among the most interesting properties that can be studied by simulation and that traditional force fields of electrolytes have never been able to reproduce correctly.^89^ In our recent work, we proposed the Madrid-Transport force field,^60,61^ which uses a scaled charge of *q* = ±0.75 and that is able to reproduce transport properties, such as the viscosities and diffusion coefficients of water and ions in the whole concentration range. This force field has also been able to reproduce transport properties in the presence of hydrogen.^90^ It should be mentioned that the introduction of scaled charges improves a number of properties of electrolytes but deteriorates the value of the free energy of solvation (although it can be corrected via theoretical corrections^85^).

In this work, we want to analyze in detail the quality of the predictions for electrical conductivities of force fields by using either integer or scaled charges. To the best of our knowledge, such a detailed comparison has never been presented before. As we will show in the next section, electrical conductivities can be calculated with the Einstein–Helfand (EH) equation.^91−97^ In a preliminary but pioneering work, Lyubartsev and Laaksonen^98^ evaluated the electrical conductivities of NaCl aqueous solutions at different concentrations with the flexible SPC model for water and ions described as charged LJ particles by using the Green–Kubo (GK) equation^99^ (which is strictly equivalent to the Einstein–Helfand relation). Due to the computational cost of evaluating the conductivities in this way,^100^ many authors tend to calculate the electrical conductivities by using the Nernst–Einstein equation (i.e., neglecting the ion–ion correlations).^101−106^ Although this is cheaper from a computational point of view, the results are not exact (as this is an approximation) and overestimate the real conductivities of the model. Electrical conductivities have been accurate and extensively calculated (through GK or EH) by different authors for ionic liquids.^91,92,107−110^ In the case of aqueous electrolyte solutions, there are a few studies in which the conductivities were properly evaluated.^93,111,112^ Martí, Guardia, and co-workers^111^ calculated electrical conductivities of NaCl solutions at different concentrations using the Smith and Dang^34^ ion force field with SPC/E water^18^ (SD-SPC/E). Shao et al.^112^ also calculated the conductivities of NaCl solutions but using in this case the Joung and Cheatham^42^ force field in combination with SPC/E water (JC-SPC/E). In fact, these authors showed interesting results about the existence of finite size effects when conductivities were calculated with the Nernst–Einstein relation. There are also semiempirical fitted models that try to reproduce the electrical conductivities of NaCl solutions in solvent mixtures, such as water–propylene carbonate^113^ or water–monoethylene glycol.^114^ Other authors have also rigorously computed electrical conductivities for molten salts.^115,116^ Nevertheless, there is no comprehensive molecular dynamics (MD) study of the performance of different force fields of ions and water for reproducing the electrical conductivities of NaCl solutions in water.

The main purpose of this work is to provide a benchmark to calculate electrical conductivities of aqueous electrolyte solutions, to show that there is a force field able to reproduce the experimental conductivities of NaCl and KCl solutions, and finally, from a deeper perspective, we want to demonstrate that to reproduce conductivities the two surfaces present in water have to be simultaneously described. We will properly evaluate the electrical conductivities of different well-known ion force fields in combination with different water force fields. Besides, in this work, we introduce a new “conceptual” strategy to determine electrical conductivities. As we have mentioned in the past, water (and aqueous solutions) has two surfaces: the potential energy surface (PES) that describes the energy of each configuration of the system and the dipole moment surface (DMS) that describe the dipole moment of each configuration.^117−119^ The key idea is that these two surfaces can be described by different fitting parameters (i.e., with different charges in our case). In this work, we use a force field with scaled charges to perform the MD simulations of aqueous electrolyte solutions. Only the PES is needed to perform these simulations as we are using the linear response theory, which evaluates transport properties (electrical conductivities in this work) by analyzing the fluctuations of the system when at equilibrium (in the absence of an electric field in the case of electrical conductivities). We will use the Madrid-Transport force field (*q* = ±0.75) for the PES as it is able to describe other properties of electrolyte solutions accurately (i.e., densities, viscosities, and diffusion coefficients). The obtained trajectories are analyzed employing unit charges to calculate the electrical conductivities (as integer charges instead of partial charges provide a better representation of the DMS). In this way, we will show how to evaluate conductivities of aqueous electrolyte solutions with a new methodology that yields reproducibility of the experimental conductivities of NaCl and KCl solutions by using a scaled charge force field.

## 2 Methodology

Electrical conductivities can be calculated using the Einstein–Helfand relation:^91−95^
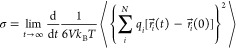
1where *V* is the system volume, *k*_B_ the Boltzmann constant, *T* the temperature, *r⃗*_*i*_(*t*) and *r⃗*_*i*_(0) the position of the *i*^*th*^ particle at time *t* and 0, respectively, and the ⟨[*r⃗*_*i*_(*t*) – *r⃗*_*i*_(0)]^2^⟩ term is the mean square displacement (MSD). Taking into account that the dipole moment of the system can be defined as
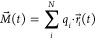
2we can obtain the following equation for the conductivity in function of the mean square dipole displacement of the system:
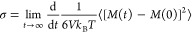
3This way, the conductivity can be easily obtained from the slope of the mean square dipole displacement versus time (in the Supporting Information, we show this plot for different initial seeds of the MD simulations).

Eq 1 can be rewritten as
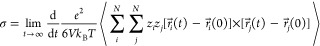
4

Please note that *q*_*i*_ is equivalent to *e*·z_*i*_. We define particles between 1 and *N*/2 as cations and particles between *N*/2 + 1 and *N* as anions. Thus, we can define for a 1:1 electrolyte the Onsager coefficients^1^ (Λ_*ij*_) for the different interactions between cations (+) and anions (−):
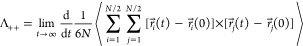
5
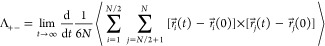
6
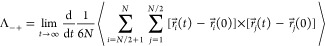
7
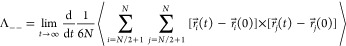
8where *N* is the total number of ions. Combining eq 4 with eqs 5–8, the electrical conductivity can be calculated from
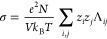
9

Eq 9 is strictly equivalent to eqs 1 and 3. Note that in all these equations, we include only the charge and positions of the ions. We do not consider water (solvent) molecules because it is a neutral molecule that does not contribute to the electrical conductivity.

Since the evaluation of electrical conductivities by the previous methodology can be computationally demanding, some authors use the approximate Nernst–Einstein (NE) equation,^120^ which relates the electrical conductivity to the self-diffusion coefficients of the ions:
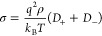
10where *q* is the charge of the ions, ρ is the number density of the salt, and *D*_+_ and *D*_–_ are the self-diffusion coefficients of the cation and anion, respectively. The NE approximation assumes that the only terms that contribute to the conductivities are those of the particle with itself. Note that whereas there are *N*^2^ terms in the rigorous expression of the conductivity (eq 4), there are only *N* terms when using the NE relation (eq 10). The NE relation thus can be obtained from eq 1 by assuming that the displacement of ions is independent (i.e., ⟨*r*_*i*_ · *r*_*j*_⟩ = 0 for *i* ≠ *j*). Making this assumption, electrical conductivities can be easily obtained for equimolar salts with eq 10. For the evaluation of conductivities with the NE equation, we have calculated the self-diffusion coefficients of both Na^+^ and Cl^–^ by using the Einstein relation (eq 11):
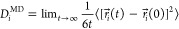
11where *r⃗*_*i*_(*t*) and *r⃗*_*i*_(0) are the position of the *i*^*th*^ particle at time *t* and 0, and the ⟨[*r⃗*_*i*_(*t*) – *r⃗*_*i*_(0)]^2^⟩ term is the MSD. All diffusivities in this work are corrected using the hydrodynamic corrections of Yeh and Hummer,^121,122^ which are described as
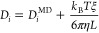
12where *D*_*i*_ is the diffusion coefficient with the applied corrections of Yeh and Hummer, *D*_*i*_^MD^ is the diffusion coefficient initially obtained by simulations, ξ is a dimensionless constant equal to 2.837, η is the computed viscosity at the studied concentration (which is shown to exhibit no finite size effects^121,123,124^), and *L* is the length of the simulation box.

## 3 Simulation Details

Electrical conductivity is not a property for which there are many previous results, and it is interesting to see whether two groups using different software packages (GROMACS vs LAMMPS), slightly different methodologies (the individual calculation of the Onsager coefficients versus the global expression), and different system sizes yield the same result for a specific model. Since we also aim to use the results of this work as a benchmark for people computing electrical conductivities in the future, and we want to ensure that the good agreement with experiment that is observed here is true regardless of the MD software or postprocessing program used for the computation. As one group is located in Madrid (UCM) and the other in Delft (TU Delft), they will be denoted as Madrid and Delft groups. Concentrations of the salts will be given in molality units, so that a solution with a concentration 1 *m* corresponds to 1 mol of salt per kilogram of water. All the results of this work correspond to room temperature and atmospheric pressure (i.e., 298.15 K and 1 bar). Note also that error bars of all the results are calculated by both groups as the standard deviation the property obtained in each run using different initial seeds divided by the square root of the number of runs.

### 3.1 Madrid Group

All MD simulations were performed with GROMACS^125,126^ (version 4.6.7). The leapfrog integrator algorithm^127^ with a time step of 2 fs was used. We also employed periodic boundary conditions in all directions. Temperature and pressure were kept constant using the Nosé–Hoover thermostat^128,129^ and Parrinello–Rahman barostat,^130^ both with a coupling constant of 2 ps. For electrostatics and van der Waals interactions, the cutoff radii were fixed at 1.0 nm, and long-range corrections in the energy and pressure were applied to the Lennard-Jones part of the potential. The smooth PME method^131^ was used to account for long-range electrostatic forces. Water geometry was maintained using the LINCS algorithm.^132,133^ To compute conductivities, we have simulated systems of 4440 water molecules and the corresponding number of ions for the desired concentration (e.g., 80 NaCl molecules for a concentration of 1 *m* as shown in Tables 1 and 2). We performed an initial *NpT* simulation of 20 ns to accurately calculate the volume of the system. After that, using the average volume obtained in the *NpT* simulation, we have carried out five independent runs in the *NVT* ensemble. Runs of 200 ns were performed for the lowest concentration (i.e., 1 *m*) and of 120 ns for the higher concentrations (2, 4, and 6 *m*). Thus, typically around 600 ns (5 × 120) or 1 μs (5 × 200) are needed to compute electrical conductivities of each model and thermodynamic state. The electrical conductivities were obtained from fitting the mean square dipole displacement (eq 2) versus time between 50 and 1000 ps, as shown in eq 3 (in the SI we also provide the results from fitting the data between 50 and 2000 ps).

**Table 1:** Computed Electrical Conductivities (σ in Units of S·m^–1^) of Aqueous NaCl Solutions from the EH Relations (Eq 3) for Different Molalities (*m* in Units of mol_salt_·kg_water_^–1^) and System Sizes^a^

	*m*	*n*_w_	*n*_s_	ρ	η	σ	σ_NE_	σ_NE+YH_
Expt.	0	–	–	997.043	0.89	0	–	–
Madrid	0	4440	0	997.3(3)	0.85(5)	0	0	0
Delft	0	1000	0	997.9(3)	0.82(1)	0	0	0
Expt	1	–	–	1036.21	0.97	8.48	–	–
Madrid	1	4440	80	1035.2(5)	0.97(7)	7.8(1)	9.7(2)	10.6(2)
Delft	1	1000	18	1034.8(1)	0.929(9)	8.7(2)	9.6(1)	11.2(1)
Delft	1	555	10	1034.9(1)	0.93(1)	8.7(2)	9.6(2)	11.5(2)
Expt.	2	–	–	1072.27	1.08	14.49	–	–
Madrid	2	4440	160	1070.3(5)	1.12(7)	14.0(1)	17.3(1)	18.9(1)
Delft	2	1000	36	1069.9(2)	1.07(2)	14.3(5)	17.3(2)	19.9(2)
Delft	2	555	20	1070.1(1)	1.08(2)	13.8(6)	16.7(1)	19.9(1)
Expt.	4	–	–	1136.91	1.35	22.04	–	–
Madrid	4	4440	320	1135.4(5)	1.44(10)	20.1(4)	27.3(1)	29.6(1)
Delft	4	1000	72	1134.92(7)	1.37(3)	20.4(8)	27.4(2)	31.3(2)
Delft	4	555	40	1135.31(9)	1.32(3)	21.6(5)	26.5(2)	31.4(1)
Expt.	6	–	–	1192.88	1.75	25.03	–	–
Madrid	6	4440	480	1194.5(5)	1.79(10)	22.2(8)	32.6(01)	35.2(01)
Delft	6	1000	108	1194.0(2)	1.69(2)	23.8(5)	32.53(7)	37.04(8)
Delft	6	555	60	1194.0(1)	1.69(1)	23.8(6)	32.2(3)	37.7(3)

a All simulations were performed at 1 bar and 298.15 K, using the Madrid-Transport model. The number of water molecules (*n*_W_) and NaCl molecules (*n*_s_), the corresponding densities (ρ in units of kg m^–3^) and viscosities (η in units of mPa·s) are shown for all molalities. Additional electrical conductivities computed using the Nernst–Einstein with (σ_NE+YH_ in units of S·m^–1^) and without (σ_NE_ in units of S·m^–1^) Yeh–Hummer finite-size corrections^121,122,124^ are reported as well. Numbers in parentheses are the uncertainty in the last digit of the results.

**Table 2:** Computed Electrical Conductivities (σ in Units of S·m^–1^) of Aqueous KCl Solutions from the EH Relations (Eq 3) for Different Molalities (*m* in Units of mol_salt_·kg_water_^–1^) and System Sizes^a^

	*m*	*n*_w_	*n*_s_	ρ	η	σ	σ_NE_	σ_NE+YH_
Expt.	0	–	–	997.043	0.89	0	–	–
Madrid	0	4440	0	997.3(3)	0.85(5)	0	0	0
Delft	0	1000	0	997.9(3)	0.82(1)	0	0	0
Expt.	2	–	–	1081.5	0.90	19.98	–	–
Madrid	2	4440	160	1081.1(5)	0.95(5)	20.4(9)	24.0(1)	25.8(2)
Delft	2	1000	36	1080.6(1)	0.91(4)	20.8(3)	23.7(2)	26.7(3)
Delft	2	555	20	1080.8(1)	0.92(2)	20.8(5)	22.8(4)	26.5(3)
Expt.	4	–	–	1152.2	0.94	34.15	–	–
Madrid	4	4440	320	1152.3(5)	1.03(7)	32.5(6)	40.5(1)	43.1(1)
Delft	4	1000	72	1151.60(5)	1.00(2)	32.9(9)	39.7(7)	44.8(7)
Delft	4	555	40	1151.81(3)	0.99(2)	32.4(7)	38.8(2)	45.1(3)

a All simulations were performed at 1 bar and 298.15 K, using the Madrid-Transport model. The number of water molecules (*n*_W_) and KCl molecules (*n*_s_), the corresponding densities (ρ in units of kg m^–3^) and viscosities (η in units of mPa·s) are shown for all molalities. Additional electrical conductivities computed using the Nernst–Einstein with (σ_NE+YH_ in units of S·m^–1^) and without (σ_NE_ in units of S·m^–1^) Yeh–Hummer finite-size corrections^121,122,124^ are reported as well. Numbers in parentheses are the uncertainty in the last digit of the results.

### 3.2 Delft Group

MD simulations are carried out on the DelftBlue supercomputer at TU Delft^134^ with the large-scale atomic/molecular massively parallel simulator (LAMMPS: version August 2018).^135^ Periodic boundary conditions are imposed in all directions, and the velocity-Verlet algorithm is used with a time step of 2 fs. The Nosé–Hoover thermostat and barostat^128−130^ are set with coupling constants of 0.1 and 1 ps, respectively. The SHAKE algorithm is used to fix the bond lengths and angles of water.^135,136^ A cutoff of 1.0 nm is used for both the Lennard-Jones and electrostatic potentials. Long-range electrostatic interactions are modeled using the particle–particle particle–mesh (PPPM) method^137,138^ with a relative error^139^ of 10^–5^. Analytic tail corrections^137^ are applied to the Lennard-Jones interactions for both energies and pressures. Initial configurations are created using PACKMOL (v20.3.1).^140^ To compute electrical conductivities, self-diffusivities, and shear viscosities, the OCTP plugin^141^ is used. In this plugin, the Einstein relations are used in combination with the order-*n* algorithm^137,142^ to compute transport properties. All details on the OCTP plugin can be found in ref (141). The approach to evaluate conductivities is based on the computation of the Onsager coefficients (Λ_*ij*_) for the cation–cation, anion–anion, and cation–anion interactions independently as shown in eqs 4–9. These equations are used to compute the exact electrical conductivities, accounting for ion–ion correlations. In the SI we have collected the results for the individual contributions to the electrical conductivities (i.e., σ_++_,σ_+–_, σ_–+_, and σ_––_) computed from each individual Onsager coefficient. To evaluate the electrical conductivities, the system sizes were 555 or 1000 water molecules (the corresponding number of ion molecules is dictated from the molality of each system), as we have listed in Tables 1 and 2. To accurately compute the average volume of the simulation box, simulations of 20 ns in the *NpT* ensemble were initially carried out (10 ns equilibration runs followed by 10 ns production runs). The self-diffusivities, viscosities, and Onsager coefficients are calculated from production runs of 200 ns in the *NVT* ensemble. Three different simulations were carried out with different initial velocities for all molalities to obtain statistics. Thus, a total simulation time of ca. 600 ns is required to compute the electrical conductivities of each model and thermodynamic state.

## 4 Results

### 4.1 Electrical Conductivities of Popular Force Fields

**Figure 1:**
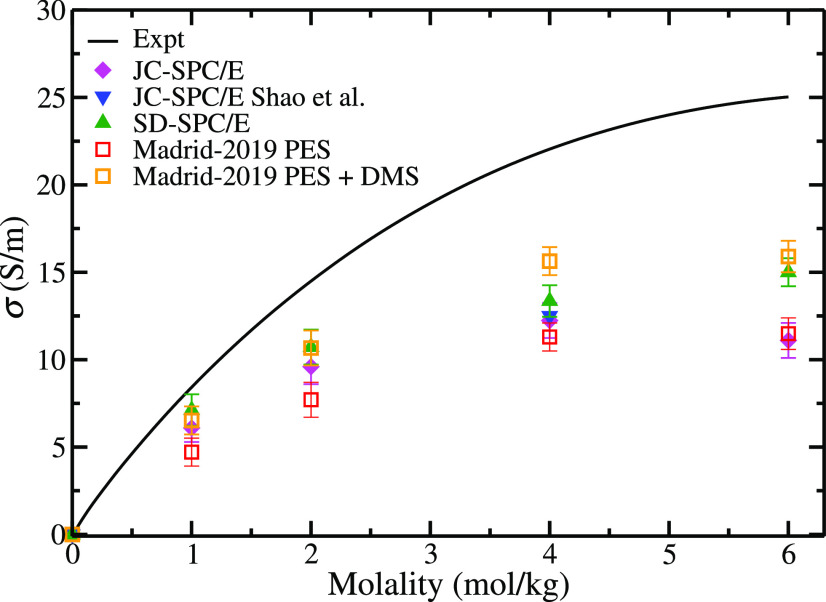
Electrical conductivities (computed by the Madrid group) as a function of NaCl concentration obtained with the different models studied in this work using eq 3 at temperature *T* = 298.15 K, and pressure *p* = 1 bar. Note that Madrid-2019 PES (red empty squares) uses scaled charges in both MD simulations and for computing the conductivities, but Madrid-2019 PES+DMS uses scaled charges for the dynamics and integer charges for computing the conductivities. Experimental results have been taken from ref (143).

In previous studies, we have shown that popular force fields (that use integer charges for the ions) are not able to reproduce transport properties, such as viscosities, diffusion coefficients of water in salt solutions, and self-diffusion coefficients of ions.^58,59,61^ Here we investigate if these models also fail in accurately predicting another important transport property, namely, the electrical conductivity. In Figure 1, results of the electrical conductivity of NaCl obtained in this work for two popular force fields (i.e., Joung and Cheatham^42^ and Smith and Dang^34^) that use integer charges combined with the SPC/E water model are compared to experiments. Contrary to previous studies that only focused on concentrations up to 4 m, in this work, we have evaluated the conductivities in the whole concentration range, i.e., up to the experimental solubility limit of NaCl (6.1 *m*). Our results at 4 *m* for the JC-SPC/E are in excellent agreement with those obtained by Shao et al.^112^ using the Green Kubo formalism. Results presented in Figure 1 were obtained from the Madrid group. It is clear that electrical conductivities are underestimated with respect to experiments by the models using integer values for the charge. The SD-SPC/E is slightly more accurate compared to the JC-SPC/E, but also underestimates the electrical conductivities. Thus, it is evident that these two popular force fields for NaCl are not able to reproduce electrical conductivities. These models overestimate the experimental viscosities of NaCl solutions.^61^ It is not surprising that electrical conductivities are underestimated, as intuitively one would expect that an overestimate of the viscosity would lead to an underestimate of the diffusion coefficient of the ions and, therefore, to an underestimate of the electrical conductivity.

### 4.2 Electrical Conductivities of Scaled Charge Models

Unit charge force fields cannot reproduce electrical conductivities of aqueous solutions, but in previous works we have demonstrated that scaled charge models significantly improve the description of transport properties as viscosities and self-diffusion coefficients.^60,61^ Thus, we have now employed the Madrid-2019 force field to observe whether we can improve the results of the unit charge force fields. This force field uses scaled charges for the ions (in particular ±0.85). In Figure 1, we show the results for the conductivities of the Madrid-2019 model (red empty squares). Surprisingly we do not obtain better results than those of the unit charge models. This was also observed by Gullbrekken et al.^144^ in their recent work in which they studied the electrical conductivities by using an integer charge force field but then when scaling the charges they did not observe differences. Regarding Madrid-2019 results, one may wonder how is it possible than a force field that yields better results for viscosities and diffusion coefficients of aqueous solutions does not perform equally well for electrical conductivity. We provide now a possible explanation for this puzzling behavior. As discussed in detail previously,^117^ water has two different surfaces, the PES and the DMS. In the absence of an external macroscopic electric field, all properties of a system can be determined in computer simulations from the PES. The DMS surface is not needed to determine any property of a system when an external electric field is not applied. However, certain properties describe the response of a system to an external electric field. In particular, the dielectric constant and electrical conductivity are response functions of this type. Obviously, these properties are relevant only when an external electric field is applied to the system. Therefore, to determine these response functions in computer simulations, it is required to describe both the PES and the DMS. A clarification is now in order. The PES simply gives the energy of a system provided the positions of all of the nuclei of the system. The PES only depends on the position of the atoms and does not depend on any macroscopic property such as the viscosity or the dielectric constant. Sometimes it is stated that the dielectric constant enters into the description of the PES in the case of electrolytes. This is not correct. Even for electrolytes, one simply needs to know the position of the atoms to determine the energy of the system, and the value of the dielectric constant is not needed. The origin of this confusion arises from the fact that at infinite dilution, and when *r* tend to ∞, the potential of mean force *w*(*r*) between two ions obtained defined from the radial distribution function *g*(*r*) using the following expression:
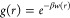
13can be obtained from the knowledge of the dielectric constant ϵ_*r*_ as follows:
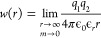
14

However, the potential of mean force is not the PES, and besides this expression is valid only in the Debye–Huckel limit (i.e., at infinite dilution of electrolyte and infinitely large distances). The summary is that the dielectric constant does not enter in the description of the PES, and in the absence of an external electric field, all properties of an aqueous electrolyte solution can be obtained from the PES and the knowledge of the DMS is not needed.

Imagine that a model can describe correctly the viscosities and the diffusion coefficients. This is an indicator that the PES is described correctly. Imagine now that the electrical conductivity is not well described. How can we solve this paradox? The answer is rather simple: the PES is well described, but the DMS is not well described. Often partial charges are used to describe the PES and these charges are also used to describe the DMS. We have suggested sometime ago that the charges that are good to describe the PES may not be suitable to describe the DMS.^117^ We have provided some indications by analyzing the behavior of the dielectric constant of water. Jorge and co-workers^119,145^ have shown that the same is true for the dielectric constant of alcohols, and Bowman and co-workers followed up on this idea.^146,147^

**Figure 2:**
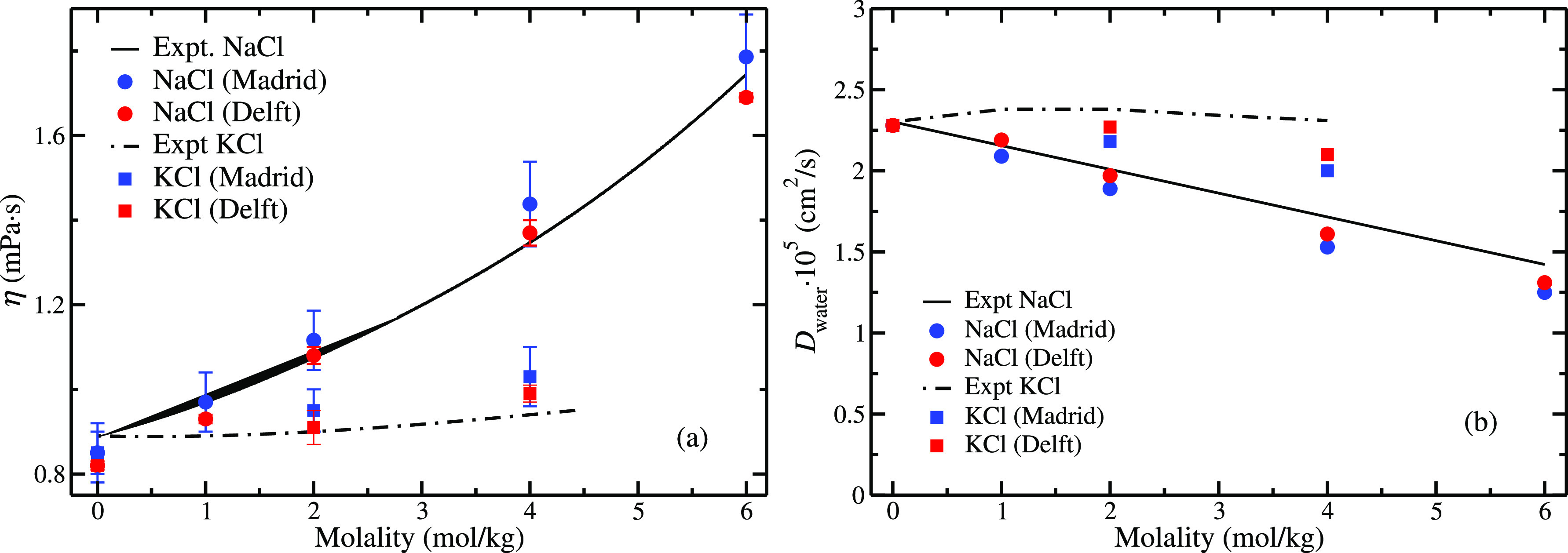
Transport properties of NaCl and KCl aqueous solutions at different concentrations obtained with the Madrid-Transport force field by Madrid (blue) and Delft (red) groups at a temperature of *T* = 298.15 K and pressure of *p* = 1 bar. (a) Viscosities and (b) self-diffusion coefficients of water (corrected for system size effects). Experimental results have been taken from refs (148 and 149).

Here, we use different charges to describe the PES and the DMS of electrolytes in water. In particular, we shall use scaled charges for the ions in water for the PES while we shall use nonscaled charges (i.e., integer charges) to describe the DMS. The way to implement this idea is rather simple. Since we are using linear response theory (so that the electrical conductivity is computed by simulating the system in the absence of the electric field), we shall perform MD simulations to obtain the trajectories using the scaled charge of the ions (i.e., ±0.85 for the particular case of the Madrid-2019 force field). In sharp contrast, when the trajectory is analyzed using eqs 1–9, integer charges are used for the ions. The results from this approach are presented for the Madrid-2019 force field in Figure 1 (orange empty squares). As clearly shown, the computed conductivities are closer to the experimental data. These results showcase that a better description is obtained when simultaneously describing both surface, the PES and DMS. However, we do not obtain a perfect agreement with the experimental electrical conductivities of the aqueous solutions. This is not entirely surprising, as the Madrid-2019 force field (that uses a scaled charge of ±0.85) improves the description of transport properties of electrolytes in water but is not able to yield quantitative agreement with experiments. To this end, we have recently proposed a force field for NaCl and KCl (denoted as Madrid-Transport) that is able to predict transport properties of these electrolyte solutions with excellent agreement to the experiment.^60,61^ Therefore, we shall compute the electrical conductivities of NaCl and KCl solutions using the Madrid-Transport force field (which uses a scaled charge of ±0.75). We shall implement the main idea of this work, namely, to use scaled charges to obtain the trajectories and integer charges to describe the DMS (i.e., using integer charges in eqs 1–9). In Figure 2, we present both properties (viscosities and self-diffusion coefficients of water) in the whole concentration range of each salt up to the experimental solubility limit. Results are independently calculated by two different research groups: Madrid (blue) and Delft (red) are consistent within the error bars. The results are in good agreement with experiments showing that these two transport properties that are obtained from the PES are described satisfactorily by the Madrid-Transport force field.

**Figure 3:**
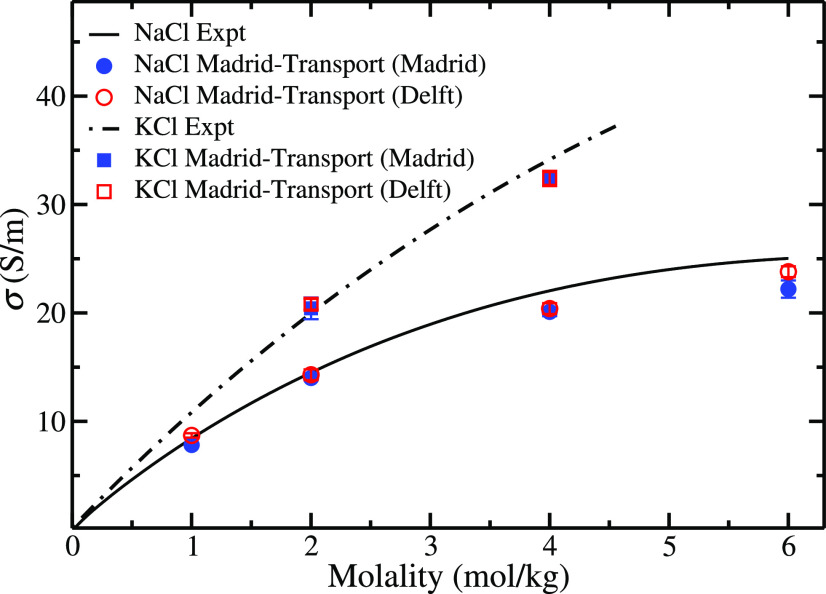
Electrical conductivities as a function of NaCl concentration obtained with the Madrid-Transport force field in independent research groups: Madrid (blue) using eq 3 and Delft (red) using eq 9, for NaCl (circles) and KCl (squares) aqueous solutions at temperature *T* = 298.15 K and pressure *p* = 1 bar. Experimental results have been taken from ref (143).

In Figure 3 the results for the electrical conductivities of NaCl and KCl solutions using the Madrid-Transport force field are shown. Note that these results are computed with our novel approach (i.e., using scaled charges *q*= ±0.75 to describe the trajectories of the system and unit charges to compute the electrical conductivities). We show the results obtained from two different research groups: Madrid (blue) and Delft (red). Both groups adopted the same approach to calculate conductivities (i.e., employing the EH equations) with some minor differences. Madrid has evaluated the conductivity from the mean square dipole displacement, taking into account all the interactions between ions (cation–cation, anion–anion, and cation–anion). Delft has evaluated the Onsager coefficients independently for the cation–cation, anion–anion, and cation–anion interactions and then summed up all the contributions. Both approaches are equivalent and thus should yield identical results. It is important to note that both groups have used different software, constraint algorithms, system sizes, simulation times, and fitting methods (see Simulation Details). Even so, the results for the conductivities obtained by both groups are equal within the error bars. Note also that despite the system sizes used by Delft and Madrid groups being different (between 555 and 4440 water molecules), the electrical conductivities are in agreement, showing that no finite size effects in the computation of electrical conductivities are observed. Nevertheless, since both self-and collective (Maxwell Stefan and Fick) diffusivities exhibit significant finite size effects,^121,122,150,151^ a thorough investigation for electric conductivities should be also performed. This is particularly important for small concentrations (i.e., below 1 *m*). This was also previously studied by Shao et al.^112^ for the JC-SPC/E model concluding that there were not finite size effects when using the Green–Kubo equation. In Figure 3, we can observe that the conductivities obtained by both groups for the Madrid-Transport force field reproduce the experimental conductivities of both NaCl and KCl aqueous solutions over the whole concentration range. Note that the electrical conductivity of KCl is significantly larger than that of NaCl at the same concentration. This difference is correctly described by the Madrid-Transport force field. Thus, the Madrid-Transport force field and the use of scaled charges for the trajectories and integer charges for computing conductivities (i.e., describing the PES with scaled charges and the DMS with integer charges) allows for the first time to correctly reproduce experimental conductivities (and other transport properties as viscosities and water diffusion coefficients). In Tables 1 and 2, we have collected the computed conductivities for each system (by both groups) along with the conductivities obtained by using the NE equation (eq 10) with and without applying the finite-size corrections to the self-diffusion coefficients.

### 4.3 Electrical Conductivities by Using the Nernst–Einstein Equation

The correct way to calculate conductivities is to use the Green–Kubo or the Einstein–Helfand equations. However, the NE equation (eq 10) is widely used due to its simplicity. In Tables 1 and 2, the electrical conductivities predicted by the approximate NE formula are also presented. As can be seen, the NE relation overestimates the true conductivity of the model, as obtained from the EH equations. Not surprisingly, the NE equation does not provide the exact value of the electrical conductivity of the model (see the Supporting Information). The reason for this is that it neglects correlations between different ions. The deviations increase with the concentration but they are clearly visible even at a concentration of 1 *m*. Our results suggest that correlations between different ions tend to decrease the value of the electrical conductivity. The conclusion from the results of Tables 1 and 2 is that NE should not be used to estimate electrical conductivities, as it provides incorrect results. If one wants to compute the true conductivity of a force field, either the GK or EH expressions should be used.

**Figure 4:**
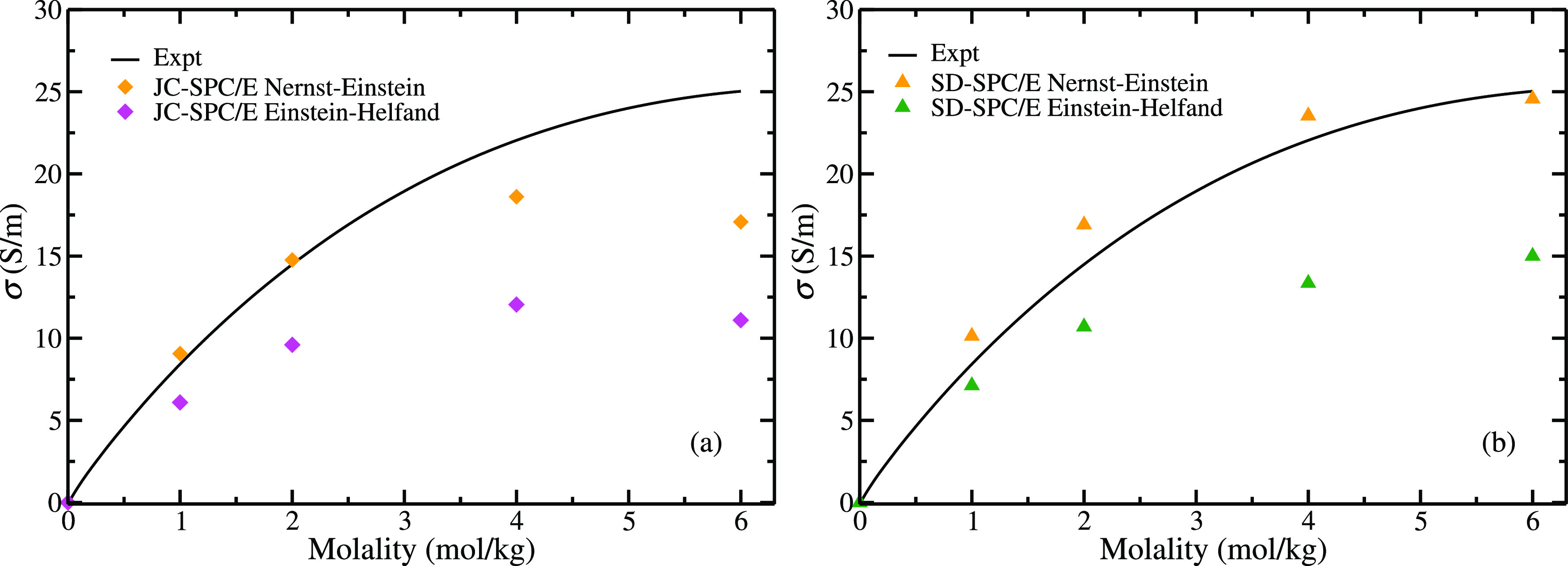
Electrical conductivities as a function of NaCl concentration obtained with different models studied in this work by two different methodologies (i.e., by using the EH relation and the Nernst–Einstein equation) at *T* = 298.15 K, *p* = 1 bar. (a) JC-SPC/E (b) SD-SPC/E. Experimental results have been taken from ref (143).

Despite being inaccurate, the NE expression is often used in many papers to obtain electrical conductivities. There are two main reasons for this. The first one is that the calculation of self-diffusion coefficients is relatively easy and computationally cheap. Thus, if the NE formalism is used, then the electrical conductivities are obtained with no additional computational cost. Diffusion coefficients have good statistics, as one can accumulate the statistics of each individual ion, while the EH is expensive, as it is a global property and each configuration contributes a single value of the correlation function. In short, diffusion coefficients of ions can be obtained with good accuracy in runs of 20 ns, whereas ca. 600 ns is needed to have reasonable statistics of the electrical conductivity. Additionally, the computation of the diffusion coefficient is implemented in many MD packages, but this is not the case for the electrical conductivity. The second reason why NE is so popular is because most of the force fields tend to underestimate significantly the electrical conductivity compared to the experiments when they are computed rigorously from the EH formalism. However, since NE electrical conductivities are much larger, they tend to be in better agreement with the experimental results. This creates the paradox that despite NE tends to overestimate the electric conductivities, in many cases, it is closer to experimental data, and thus, there is a resistance to abandon its use. Obviously, this apparent agreement arises from a cancellation of errors (i.e., a poor force field along with a poor way of computing the actual conductivity of the force field can provide good agreement with experiments). This is illustrated in Figure 4, where the electrical conductivities of the JC-SPC/E and SD-SPC/E obtained from NE and from EH are compared to the experimental. As it can be seen, the agreement is better with NE. As we have shown, NE does not describe correctly the electrical conductivity of the force field so that the apparent improvement is obtained from fortuitous cancellation of two errors (the force field and the way to compute the electrical conductivity). Another “apparent” advantage of the NE formalism is that since diffusion coefficients are quite sensitive to the size of the system, one can often find a system size for which the agreement with experiment is excellent. This adds another degree of freedom for “fine-tuning” the final value of the electric conductivity. However, this should not be accepted when striving for an accurate computation of properties. This becomes even more pronounced by the fact that the system size dependency of the electrical conductivity when computed from the EH formalism is quite small as it has been shown in this work (i.e., compare the results from Madrid and from Delft) and also shown in other works.^112^

**Figure 5:**
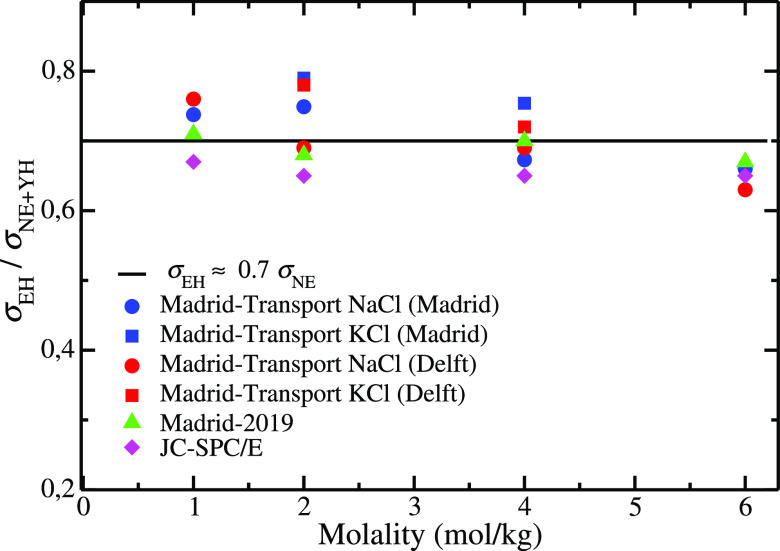
Ratio between the conductivities obtained by using the Einstein–Helfand formula (σ_EH_) and the Nernst–Einstein equation (σ_NE+YH_).

After this work, we strongly advise against using NE when one can actually compute the conductivity using rigorous ways. NE can be used when one has no access to such a computation or when a quick estimation of the order of magnitude of the conductivity is needed. However, even in such cases, the researcher should keep in mind that the NE value is overestimated with respect to the real conductivity. However, we suggest an approximate (and not rigorous) way of at least correcting NE results. The idea is simple. We have analyzed the typical ratio between the electrical conductivities obtained rigorously (i.e., EH relation) and those obtained from the approximate NE formalism and have analyzed whether it is approximately constant for different force fields and concentrations. When computing the electrical conductivity from NE, we used the finite size corrected diffusion coefficients. In this way, the NE conductivities computed here are determined using one of the most correct approaches to estimate diffusion coefficients in the thermodynamic limit. In Figure 5, we show the ratio between the conductivities obtained by EH divided by the conductivities calculated with NE as a function of the concentration. Notice that for models with different charges (JC-SPC/E, Madrid-2019 and Madrid-Transport), for all concentrations and even for both studied salts (NaCl and KCl), the conductivities using the EH equation are about 30% lower than those when using the NE equation. Thus, a rough approximation of the EH conductivities can be obtained simply by employing:
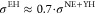
15where σ^EH^ is the conductivity rigorously computed by using the EH relation, and σ^NE+YH^ is the approximate conductivity calculated with the NE equation (after including Yeh–Hummer corrections to the values of the diffusion coefficients). If one wants to roughly estimate the correct conductivities of a model (i.e., evaluated by the EH relation), one can employ only the NE equation and then apply our rule described in eq 15. In this way, one can obtain an approximate (with a typical error of about 5–8%) but still reasonable estimate of the true conductivity of the force field under consideration from the initial guess provided by the NE relation. In fact, regarding the recent work from Gullbrekken et al.,^144^ this rule also works properly for their results. Note also that this scaling factor in eq 15 (0.7) is empirical and is not related to the charge of ions in the force field. In any case, we recommend to evaluate properly the conductivities by using the GK or EH relations without any approximation to obtain rigorously the correct conductivity of the force field.

## 5 Conclusions

In this work, we evaluated the electrical conductivities of NaCl and KCl aqueous solutions up to their solubility limit by using the Einstein–Helfand equation. We have computed the electrical conductivities of four different NaCl force fields (i.e., JC-SPC/E, SD-SPC/E, Madrid-2019, and Madrid-Transport). We have shown that force fields with integer charges are not able to reproduce electrical conductivities, as they underestimate them considerably. Note that for these models integer charges are used to describe both the PES and the DMS. We tested a new approach that is based on the more general idea that different charges should be used to reproduce correctly the PES and the DMS. To implement this idea, we used the Madrid-Transport force field for NaCl and KCl (with scaled charges of ±0.75 for the ions) to obtain the trajectories of the system in the absence of an external electric field. The EH equation is used with integer charges for the ions. The basic idea is that scaled charges describe the PES better, and integer charges describe the DMS more accurately, as was suggested some time ago.^117,119^ By using this approach, we have shown that the Madrid-Transport force field (a model that provides excellent results for transport properties such as viscosities and diffusion coefficients) reproduces the electrical conductivities of NaCl and KCl for the whole concentration range. Certainly, one can argue that it is possible to use a model with *q* = ±1 to describe the PES and with *q* higher than ±1 for describing the DMS. By using this idea (developed in this work) the agreement with experiments for electrical conductivities will also improve for models that use integer charges for the PES. However, the cost of that is that the transported charge will not be 1 *e*, a result that has no physical meaning. In addition, the force field with that charge would not reproduce the viscosities and self-diffusion coefficients of water as in the rest of the unit charge models. It seems therefore that to reproduce the DMS one should use the integer charge that actually is transported (1 *e*), but in contrast, for describing the PES, one can use scaled charges to better describe the relative weight of some configurations with respect to others. What does quantum chemistry say about the value of the charge that is indeed transported? Is it correct to assume formal integer charges when computing electrical conductivities? This issue has been discussed in two important papers.^115,152^ For simplicity we shall discuss this issue using the Green–Kubo formalism,^153,154^ but one could also use the totally equivalent Helfand–Einstein relation. The electrical conductivity can be rigorously computed from ab initio calculations using the following expression:
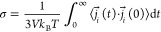
16

where *j⃗*_*i*_ is the current density vector, which is defined as
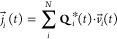
17

Apparently everything seems normal, but now comes the first surprise. The charge **Q**_i_^*^(t) is not a scalar but a time dependent tensor with components given by
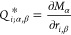
18where α, β = *x*, *y*, *z* and r_*i*,β_ refers to the component β of the vector defining the position of ion *i* and *M*_α_ is the α component of the dipole moment of the system. It should not come as a surprise that the value of the charge of the ion for the calculation of the conductivity comes from derivatives of the dipole moment of the system, which is well-defined and not from arbitrary schemes partitioning the electronic density among the different ions of the system (as for instance the Bader method^155,156^). However, it has been shown^115,152^ that one can obtain the rigorous value of the conductivities using a nontime dependent scalar for the charge of ion *i* (usually denoted as the topological charge *q*_*i*,top_). This is summarized in the following equations:
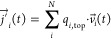
19
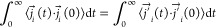
20

The conclusion is that although the charge of an ion is a time dependent tensor, there is a nontime dependent scalar value of the charge (denoted as the topological charge) that leads to the correct value of the electrical conductivity. Finally, Grasselli and Baroni,^115^ and French et al.^152^ have shown that the value of the topological charge is just the value of the formal charge. Quantum chemistry supports the use of a formal integer charge (i.e., 1 *e* for monovalent ions) in the calculation of the electrical conductivity. One should not use different values of the formal charge when computing electrical conductivities. That is what we have done in this work. However, quantum chemistry does not say anything about the charge that better fits the PES as it should be regarded as a fitting parameter of the force field, and we have shown that for aqueous electrolyte solutions, the scaled charge with value 0.75 *e* provides an excellent description of transport properties.

Finally, we have shown that when using the Nernst–Einstein equation, the electrical conductivities are incorrectly calculated due to the neglect of the correlation between different ions, showing discrepancies even at low concentrations. We propose a rule of thumb to obtain a rough estimate of the EH conductivities. The recipe is simple. It consists of multiplying by 0.7 the conductivity obtained from the NE equation (after applying YH corrections to the values of the diffusion coefficients of the individual ions). Nevertheless, this is an approximate correction, and our advice is to use the correct expression (i.e., EH or GK) to obtain rigorously the electrical conductivity of a certain force field.

The results presented in this work independently obtained by two research groups could be useful in the future as benchmark results to be reproduced by groups interested in computing the electrical conductivities of electrolyte solutions. The success of the Madrid-Transport force field in reproducing the transport properties of NaCl and KCl solutions can be regarded as a work-case example showing how fruitful the idea of using different charges to describe the PES and DMS can be in the future. The community performing classical simulations should benefit from this “mental” flexibility. In fact, the community performing ab initio calculations is already using them in an effective way as they are using different fitting parameters when developing neural networks for the PES and the DMS.^157,158^ Why should we not realize that we can do the same with our force fields?

## Data Availability

The data that support the findings of this study are available within the article and in the SI.

## Supporting Information Available

Mean square dipole displacement in function of time for different seeds of an aqueous NaCl 4 *m* solution at 298.15 K and 1 bar by using the Madrid-Transport force field, results for the self-diffusion coefficients of Na^+^ and Cl^+^ and electrical conductivities calculated with the Nernst–Einstein approximation and by using the Einstein–Helfand equation, computed electrical conductivities of aqueous NaCl solutions from the EH relations for different molalities, computed electrical conductivities of aqueous KCl solutions from the EH relations for different molalities, contributions to the electrical conductivities computed by the individual Onsager coefficients of aqueous NaCl solutions from the EH relations for different molalities, and contributions to the electrical conductivities computed by the individual Onsager coefficients of aqueous NaCl solutions from the EH relations for different molalities (PDF)

## References

[ref1] Robinson R. A.; Stokes R. H. Electrolyte solutions; Courier Corporation, 2002.

[ref2] Attias R.; Salama M.; Hirsch B.; Goffer Y.; Aurbach D. Anode-electrolyte interfaces in secondary magnesium batteries. Joule 2019, 3, 27–52. 10.1016/j.joule.2018.10.028.

[ref3] Logan E.; Dahn J. Electrolyte design for fast-charging Li-ion batteries. Trends in Chemistry 2020, 2, 354–366. 10.1016/j.trechm.2020.01.011.

[ref4] Li M.; Wang C.; Chen Z.; Xu K.; Lu J. New concepts in electrolytes. Chem. Rev. 2020, 120, 6783–6819. 10.1021/acs.chemrev.9b00531. 32022546

[ref5] Srimuk P.; Su X.; Yoon J.; Aurbach D.; Presser V. Charge-transfer materials for electrochemical water desalination, ion separation and the recovery of elements. Nature Reviews Materials 2020, 5, 517–538. 10.1038/s41578-020-0193-1.

[ref6] Enderby J.; Neilson G. The structure of electrolyte solutions. Rep. Prog. Phys. 1981, 44, 593 10.1088/0034-4885/44/6/001.

[ref7] Barthel J. M.; Krienke H.; Kunz W. Physical chemistry of electrolyte solutions: modern aspects; Springer Science & Business Media, 1998; Vol. 5.

[ref8] Kirkwood J. G. On the theory of strong electrolyte solutions. J. Chem. Phys. 1934, 2, 767–781. 10.1063/1.1749393.

[ref9] Pitzer K. S.; Press C. Activity coefficients in electrolyte solutions; CRC Press: Boca Raton, FL, 1991; Vol. 2.

[ref10] Sangster M.; Dixon M. Interionic potentials in alkali halides and their use in simulations of the molten salts. Adv. Phys. 1976, 25, 247–342. 10.1080/00018737600101392.

[ref11] Adams M. E.; McDonald I. R.; Singer K. Collective dynamical properties of molten salts: molecular dynamics calculations on sodium chloride. Proc. R. Soc. London A 1977, 357, 37–57. 10.1098/rspa.1977.0154.

[ref12] Heinzinger K.; Vogel P. A Molecular Dynamics Study of Aqueous Solutions I. First Results for LiCl in H20. Z. Naturforsch sect. A 1974, 29, 1164–1171. 10.1515/zna-1974-0809.

[ref13] Vogel P.; Heinzinger K. A Molecular Dynamics Study of Aqueous Solutions II. Cesium Chloride in H2O. Z. Naturforsch sect. A 1975, 30, 789–796. 10.1515/zna-1975-6-711.

[ref14] Heinzinger K.; Vogel P. A Molecular Dynamics Study of Aqueous Solutions. III. A Comparison of Selected Alkali Halides. Z. Naturforsch sect. A 1976, 31, 463–475. 10.1515/zna-1976-0508.

[ref15] Bernal J. D.; Fowler R. H. A Theory of Water and Ionic Solutions, with Particular Reference to Hydrogen and Hydroxyl Ions. J. Chem. Phys. 1933, 1, 515 10.1063/1.1749327.

[ref16] Jorgensen W. L.; Chandrasekhar J.; Madura J. D.; Impey R. W.; Klein M. L. Comparison of Simple Potential Functions for Simulating Liquid Water. J. Chem. Phys. 1983, 79, 926–935. 10.1063/1.445869.

[ref17] Mahoney M.; Jorgensen W. L. Quantum, intramolecular flexibility, and polarizability effects on the reproduction of the density anomaly of liquid water by simple potential functions. J. Chem. Phys. 2001, 115, 10758–10768. 10.1063/1.1418243.

[ref18] Berendsen H. J. C.; Grigera J. R.; Straatsma T. P. The missing term in effective pair potentials. J. Phys. Chem. 1987, 91, 6269–6271. 10.1021/j100308a038.

[ref19] Horn H. W.; Swope W. C.; Pitera J. W.; Madura J. D.; Dick T. J.; Hura G. L.; Head-Gordon T. Development of an improved four-site water model for biomolecular simulations: TIP4P-Ew. J. Chem. Phys. 2004, 120, 9665–9678. 10.1063/1.1683075. 15267980

[ref20] Abascal J. L. F.; Vega C. A general purpose model for the condensed phases of water: TIP4P/2005. J. Chem. Phys. 2005, 123, 234505 10.1063/1.2121687. 16392929

[ref21] Vega C.; Abascal J. L. F. Simulating water with rigid non-polarizable models: a general perspective. Phys. Chem. Chem. Phys. 2011, 13, 19663–19688. 10.1039/c1cp22168j. 21927736

[ref22] Tazi S.; Boţan A.; Salanne M.; Marry V.; Turq P.; Rotenberg B. Diffusion coefficient and shear viscosity of rigid water models. J. Phys.: Condens. Matter 2012, 24, 284117 10.1088/0953-8984/24/28/284117. 22739097

[ref23] González M. A.; Abascal J. L. F. The shear viscosity of rigid water models. J. Chem. Phys. 2010, 132, 096101 10.1063/1.3330544. 20210414

[ref24] Blazquez S.; Vega C. Melting points of water models: Current situation. J. Chem. Phys. 2022, 156, 216101 10.1063/5.0093815. 35676134

[ref25] Jiang H.; Moultos O. A.; Economou I. G.; Panagiotopoulos A. Z. Hydrogen-bonding polarizable intermolecular potential model for water. J. Phys. Chem. B 2016, 120, 12358–12370. 10.1021/acs.jpcb.6b08205. 27807969

[ref26] Reddy S. K.; Straight S. C.; Bajaj P.; Huy Pham C.; Riera M.; Moberg D. R.; Morales M. A.; Knight C.; Götz A. W.; Paesani F. On the accuracy of the MB-pol many-body potential for water: Interaction energies, vibrational frequencies, and classical thermodynamic and dynamical properties from clusters to liquid water and ice. J. Chem. Phys. 2016, 145, 194504 10.1063/1.4967719. 27875875

[ref27] Kiss P. T.; Baranyai A. A systematic development of a polarizable potential of water. J. Chem. Phys. 2013, 138, 204507 10.1063/1.4807600. 23742493

[ref28] Smith W. R.; Nezbeda I.; Kolafa J.; Moučka F. Recent progress in the molecular simulation of thermodynamic properties of aqueous electrolyte solutions. Fluid Phase Equilib. 2018, 466, 19–30. 10.1016/j.fluid.2018.03.006.

[ref29] Chandrasekhar J.; Spellmeyer D. C.; Jorgensen W. L. Energy component analysis for dilute aqueous solutions of lithium (1+), sodium (1+), fluoride (1-), and chloride (1-) ions. J. Am. Chem. Soc. 1984, 106, 903–910. 10.1021/ja00316a012.

[ref30] Straatsma T.; Berendsen H. Free energy of ionic hydration: Analysis of a thermodynamic integration technique to evaluate free energy differences by molecular dynamics simulations. J. Chem. Phys. 1988, 89, 5876–5886. 10.1063/1.455539.

[ref31] Aqvist J. Ion-water interaction potentials derived from free energy perturbation simulations. J. Phys. Chem. 1990, 94, 8021–8024. 10.1021/j100384a009.

[ref32] Dang L. X. Development of nonadditive intermolecular potentials using molecular dynamics: solvation of Li^+^ and F^−^ ions in polarizable water. J. Chem. Phys. 1992, 96, 6970–6977. 10.1063/1.462555.

[ref33] Beglov D.; Roux B. Finite representation of an infinite bulk system: solvent boundary potential for computer simulations. J. Chem. Phys. 1994, 100, 9050–9063. 10.1063/1.466711.

[ref34] Smith D. E.; Dang L. X. Computer simulations of NaCl association in polarizable water. J. Chem. Phys. 1994, 100, 3757–3766. 10.1063/1.466363.

[ref35] Roux B. Valence selectivity of the gramicidin channel: a molecular dynamics free energy perturbation study. Biophys. J. 1996, 71, 3177–3185. 10.1016/S0006-3495(96)79511-5. 8968588 PMC1233806

[ref36] Peng Z.; Ewig C. S.; Hwang M.-J.; Waldman M.; Hagler A. T. Derivation of class II force fields. 4. van der Waals parameters of Alkali metal cations and Halide anions. J. Phys. Chem. A 1997, 101, 7243–7252. 10.1021/jp964080y.

[ref37] Weerasinghe S.; Smith P. E. A Kirkwood–Buff derived force field for sodium chloride in water. J. Chem. Phys. 2003, 119, 11342–11349. 10.1063/1.1622372.

[ref38] Jensen K. P.; Jorgensen W. L. Halide, ammonium, and alkali metal ion parameters for modeling aqueous solutions. J. Chem. Theory Comput. 2006, 2, 1499–1509. 10.1021/ct600252r. 26627020

[ref39] Lamoureux G.; Roux B. Absolute hydration free energy scale for alkali and halide ions established from simulations with a polarizable force field. J. Phys. Chem. B 2006, 110, 3308–3322. 10.1021/jp056043p. 16494345

[ref40] Alejandre J.; Hansen J.-P. Ions in water: From ion clustering to crystal nucleation. Phys. Rev. E 2007, 76, 061505 10.1103/PhysRevE.76.061505. 18233853

[ref41] Lenart P. J.; Jusufi A.; Panagiotopoulos A. Z. Effective potentials for 1:1 electrolyte solutions incorporating dielectric saturation and repulsive hydration. J. Chem. Phys. 2007, 126, 044509 10.1063/1.2431169. 17286489

[ref42] Joung I. S.; Cheatham T. E. Determination of alkali and halide monovalent Ion Parameters for Use in Explicit Solvated Biomolecular Simulation. J. Phys. Chem. B 2008, 112, 9020 10.1021/jp8001614. 18593145 PMC2652252

[ref43] Corradini D.; Rovere M.; Gallo P. A route to explain water anomalies from results on an aqueous solution of salt. J. Chem. Phys. 2010, 132, 134508 10.1063/1.3376776. 20387942

[ref44] Callahan K. M.; Casillas-Ituarte N. N.; Roeselová M.; Allen H. C.; Tobias D. J. Solvation of magnesium dication: molecular dynamics simulation and vibrational spectroscopic study of magnesium chloride in aqueous solutions. J. Phys. Chem. A 2010, 114, 5141–5148. 10.1021/jp909132a. 20201546

[ref45] Yu H.; Whitfield T. W.; Harder E.; Lamoureux G.; Vorobyov I.; Anisimov V. M.; MacKerell A. D. Jr; Roux B. Simulating monovalent and divalent ions in aqueous solution using a Drude polarizable force field. J. Chem. Theory Comput. 2010, 6, 774–786. 10.1021/ct900576a. 20300554 PMC2838399

[ref46] Reif M. M.; Hünenberger P. H. Computation of methodology-independent single-ion solvation properties from molecular simulations. IV. Optimized Lennard-Jones interaction parameter sets for the alkali and halide ions in water. J. Chem. Phys. 2011, 134, 144104 10.1063/1.3567022. 21495739

[ref47] Gee M. B.; Cox N. R.; Jiao Y.; Bentenitis N.; Weerasinghe S.; Smith P. E. A Kirkwood-Buff derived force field for aqueous alkali halides. J. Chem. Theory Comput. 2011, 7, 1369–1380. 10.1021/ct100517z. 21789033 PMC3141341

[ref48] Benavides A. L.; Portillo M. A.; Chamorro V. C.; Espinosa J. R.; Abascal J. L. F.; Vega C. A potential model for sodium chloride solutions based on the TIP4P/2005 water model. J. Chem. Phys. 2017, 147, 104501 10.1063/1.5001190. 28915761

[ref49] Deublein S.; Vrabec J.; Hasse H. A set of molecular models for alkali and halide ions in aqueous solution. J. Chem. Phys. 2012, 136, 084501 10.1063/1.3687238. 22380047

[ref50] Mao A. H.; Pappu R. V. Crystal lattice properties fully determine short-range interaction parameters for alkali and halide ions. J. Chem. Phys. 2012, 137, 064104 10.1063/1.4742068. 22897252

[ref51] Mamatkulov S.; Fyta M.; Netz R. R. Force fields for divalent cations based on single-ion and ion-pair properties. J. Chem. Phys. 2013, 138, 024505 10.1063/1.4772808. 23320702

[ref52] Moučka F.; Nezbeda I.; Smith W. R. Molecular force field development for aqueous electrolytes: 1. Incorporating appropriate experimental data and the inadequacy of simple electrolyte force fields based on Lennard-Jones and point charge interactions with Lorentz–Berthelot rules. J. Chem. Theory Comput. 2013, 9, 5076–5085. 10.1021/ct4006008. 26583422

[ref53] Kiss P. T.; Baranyai A. A new polarizable force field for alkali and halide ions. J. Chem. Phys. 2014, 141, 114501 10.1063/1.4895129. 25240358

[ref54] Kolafa J. Solubility of NaCl in water and its melting point by molecular dynamics in the slab geometry and a new BK3-compatible force field. J. Chem. Phys. 2016, 145, 204509 10.1063/1.4968045. 27908102

[ref55] Elfgen R.; Hülsmann M.; Krämer A.; Köddermann T.; Kirschner K. N.; Reith D. Optimized atomistic force fields for aqueous solutions of Magnesium and Calcium Chloride: Analysis, achievements and limitations. Eur. Phys. J. Spec. Top. 2016, 225, 1391–1409. 10.1140/epjst/e2016-60112-7.

[ref56] Pethes I. A comparison of classical interatomic potentials applied to highly concentrated aqueous lithium chloride solutions. J. Mol. Liq. 2017, 242, 845–858. 10.1016/j.molliq.2017.07.076.

[ref57] Yagasaki T.; Matsumoto M.; Tanaka H. Lennard-Jones parameters determined to reproduce the solubility of NaCl and KCl in SPC/E, TIP3P, and TIP4P/2005 water. J. Chem. Theory Comput. 2020, 16, 2460 10.1021/acs.jctc.9b00941. 32207974

[ref58] Zeron I. M.; Abascal J. L. F.; Vega C. A force field of Li^+^, Na., K^+^, Mg^2+^, Ca^2+^, Cl^–^ and SO_4_^2–^ in aqueous solution based on the TIP4P/2005 water model and scaled charges for the ions. J. Chem. Phys. 2019, 151, 134504 10.1063/1.5121392. 31594349

[ref59] Blazquez S.; Conde M. M.; Abascal J. L. F.; Vega C. The Madrid-2019 force field for electrolytes in water using TIP4P/2005 and scaled charges: Extension to the ions F^–^, Br^–^, I^–^, Rb^+^, and Cs^+^. J. Chem. Phys. 2022, 156, 044505 10.1063/5.0077716. 35105066

[ref60] Habibi P.; Rahbari A.; Blazquez S.; Vega C.; Dey P.; Vlugt T. J. H.; Moultos O. A. A New Force Field for OH^–^ for Computing Thermodynamic and Transport Properties of H_2_ and O_2_ in Aqueous NaOH and KOH Solutions. J.Phys.Chem.B 2022, 126, 9376 10.1021/acs.jpcb.2c06381. 36325986 PMC9677430

[ref61] Blazquez S.; Conde M. M.; Vega C. Scaled charges for ions: and improvement but not the final word for modeling electrolytes in water. J. Chem. Phys. 2023, 158, 054505 10.1063/5.0136498. 36754806

[ref62] Panagiotopoulos A. Z. Simulations of activities, solubilities, transport properties, and nucleation rates for aqueous electrolyte solutions. J. Chem. Phys. 2020, 153, 010903 10.1063/5.0012102. 32640801

[ref63] Leontyev I. V.; Stuchebrukhov A. A. Electronic continuum model for molecular dynamics simulations. J. Chem. Phys. 2009, 130, 085102 10.1063/1.3060164. 19256627 PMC3910273

[ref64] Leontyev I. V.; Stuchebrukhov A. A. Electronic polarizability and the effective pair potentials of water. J. Chem. Theory Comput. 2010, 6, 3153–3161. 10.1021/ct1002048. 25383062 PMC4224308

[ref65] Leontyev I. V.; Stuchebrukhov A. A. Electronic continuum model for molecular dynamics simulations of biological molecules. J. Chem. Theory Comput. 2010, 6, 1498–1508. 10.1021/ct9005807. 25364313 PMC4213183

[ref66] Leontyev I. V.; Stuchebrukhov A. A. Accounting for electronic polarization in non-polarizable force fields. Phys. Chem. Chem. Phys. 2011, 13, 2613–2626. 10.1039/c0cp01971b. 21212894

[ref67] Leontyev I. V.; Stuchebrukhov A. A. Polarizable mean-field model of water for biological simulations with AMBER and CHARMM force fields. J. Chem. Theory Comput. 2012, 8, 3207–3216. 10.1021/ct300011h. 25580096 PMC4285689

[ref68] Leontyev I. V.; Stuchebrukhov A. A. Polarizable molecular interactions in condensed phase and their equivalent nonpolarizable models. J. Chem. Phys. 2014, 141, 014103 10.1063/1.4884276. 25005273 PMC4106032

[ref69] Kann Z.; Skinner J. A scaled-ionic-charge simulation model that reproduces enhanced and suppressed water diffusion in aqueous salt solutions. J. Chem. Phys. 2014, 141, 104507 10.1063/1.4894500. 25217937

[ref70] Pluhařová E.; Mason P. E.; Jungwirth P. Ion pairing in aqueous lithium salt solutions with monovalent and divalent counter-anions. J. Phys. Chem. A 2013, 117, 11766–11773. 10.1021/jp402532e. 23581250

[ref71] Kohagen M.; Mason P. E.; Jungwirth P. Accurate description of calcium solvation in concentrated aqueous solutions. J. Phys. Chem. B 2014, 118, 7902–7909. 10.1021/jp5005693. 24802184

[ref72] Kohagen M.; Mason P. E.; Jungwirth P. Accounting for electronic polarization effects in aqueous sodium chloride via molecular dynamics aided by neutron scattering. J. Phys. Chem. B 2016, 120, 1454–1460. 10.1021/acs.jpcb.5b05221. 26172524

[ref73] Duboué-Dijon E.; Mason P. E.; Fischer H. E.; Jungwirth P. Hydration and ion pairing in aqueous Mg^2+^ and Zn^2+^ solutions: force-field description aided by neutron scattering experiments and ab initio molecular dynamics simulations. J. Phys. Chem. B 2018, 122, 3296–3306. 10.1021/acs.jpcb.7b09612. 29116789

[ref74] Martinek T.; Duboué-Dijon E.; Timr v.; Mason P. E.; Baxová K.; Fischer H. E.; Schmidt B.; Pluhařová E.; Jungwirth P. Calcium ions in aqueous solutions: Accurate force field description aided by ab initio molecular dynamics and neutron scattering. J. Chem. Phys. 2018, 148, 222813 10.1063/1.5006779. 29907056

[ref75] Fuentes-Azcatl R.; Barbosa M. C. Sodium chloride, nacl/ε: New force field. J. Phys. Chem. B 2016, 120, 2460–2470. 10.1021/acs.jpcb.5b12584. 26890321

[ref76] Fuentes-Azcatl R.; Barbosa M. C. Potassium bromide, KBr/ε: New Force Field. Phys. A 2018, 491, 480–489. 10.1016/j.physa.2017.09.081.

[ref77] Li J.; Wang F. Pairwise-additive force fields for selected aqueous monovalent ions from adaptive force matching. J. Chem. Phys. 2015, 143, 194505 10.1063/1.4935599. 26590540 PMC4654740

[ref78] Bruce E. E.; van der Vegt N. F. A. Does an electronic continuum correction improve effective short-range ion-ion interactions in aqueous solution?. J. Chem. Phys. 2018, 148, 222816 10.1063/1.5017101. 29907065

[ref79] Yao Y.; Berkowitz M. L.; Kanai Y. Communication: Modeling of concentration dependent water diffusivity in ionic solutions: Role of intermolecular charge transfer. J. Chem. Phys. 2015, 143, 241101 10.1063/1.4938083. 26723580

[ref80] Soniat M.; Rick S. W. The effects of charge transfer on the aqueous solvation of ions. J. Chem. Phys. 2012, 137, 044511 10.1063/1.4736851. 22852635

[ref81] Lee A. J.; Rick S. W. The effects of charge transfer on the properties of liquid water. J. Chem. Phys. 2011, 134, 184507 10.1063/1.3589419. 21568521

[ref82] Soniat M.; Rick S. W. Charge transfer effects of ions at the liquid water/vapor interface. J. Chem. Phys. 2014, 140, 184703 10.1063/1.4874256. 24832295

[ref83] Soniat M.; Pool G.; Franklin L.; Rick S. W. Ion association in aqueous solution. Fluid Phase Equilib. 2016, 407, 31–38. 10.1016/j.fluid.2015.05.001.

[ref84] Le Breton G.; Joly L. Molecular modeling of aqueous electrolytes at interfaces: Effects of long-range dispersion forces and of ionic charge rescaling. J. Chem. Phys. 2020, 152, 241102 10.1063/5.0011058. 32610967

[ref85] Blazquez S.; Zeron I. M.; Conde M. M.; Abascal J. L. F.; Vega C. Scaled charges at work: Salting out and interfacial tension of methane with electrolyte solutions from computer simulations. Fluid Phase Equilib. 2020, 513, 112548 10.1016/j.fluid.2020.112548.

[ref86] Sedano L. F.; Blazquez S.; Noya E. G.; Vega C.; Troncoso J. Maximum in density of electrolyte solutions: Learning about ion-water interactions and testing the Madrid-2019 force field. J. Chem. Phys. 2022, 156, 154502 10.1063/5.0087679. 35459318

[ref87] Lamas C. P.; Vega C.; Noya E. G. Freezing point depression of salt aqueous solutions using the Madrid-2019 model. J. Chem. Phys. 2022, 156, 134503 10.1063/5.0085051. 35395902

[ref88] Zeron I. M.; Gonzalez M. A.; Errani E.; Vega C.; Abascal J. L. F. “In Silico” Seawater. J. Chem. Theory Comput. 2021, 17, 1715–1725. 10.1021/acs.jctc.1c00072. 33533631

[ref89] Kim J. S.; Wu Z.; Morrow A. R.; Yethiraj A.; Yethiraj A. Self-diffusion and viscosity in electrolyte solutions. J. Phys. Chem. B 2012, 116, 12007–12013. 10.1021/jp306847t. 22967241

[ref90] van Rooijen W.; Habibi P.; Xu K.; Dey P.; Vlugt T. J. H.; Hajibeygi H.; Moultos O. Interfacial Tensions, Solubilities, and Transport Properties of the H_2_/H_2_O/NaCl System: A Molecular Simulation Study. J. Chem. Eng. Data 2023, 10.1021/acs.jced.2c00707. PMC10859954 38352074

[ref91] Schröder C.; Haberler M.; Steinhauser O. On the computation and contribution of conductivity in molecular ionic liquids. J. Chem. Phys. 2008, 128, 134501 10.1063/1.2868752. 18397071

[ref92] Picálek J.; Kolafa J. Molecular dynamics study of conductivity of ionic liquids: The Kohlrausch law. J. Mol. Liq. 2007, 134, 29–33. 10.1016/j.molliq.2006.12.015.

[ref93] Nieszporek K.; Nieszporek J.; Trojak M. Calculations of shear viscosity, electric conductivity and diffusion coefficients of aqueous sodium perchlorate solutions from molecular dynamics simulations. Computational and Theoretical Chemistry 2016, 1090, 52–57. 10.1016/j.comptc.2016.06.002.

[ref94] Helfand E. Transport coefficients from dissipation in a canonical ensemble. Phys. Rev. 1960, 119, 1 10.1103/PhysRev.119.1.

[ref95] Malaspina D. C.; Lisal M.; Larentzos J. P.; Brennan J. K.; Mackie A.; Avalos J. B. Transport coefficients from Einstein-Helfand relations using standard and energy-conserving dissipative particle dynamics methods. Phys. Chem. Chem. Phys. 2023, 25, 12025 10.1039/D2CP04838H. 37082893

[ref96] Celebi A. T.; Vlugt T. J. H.; Moultos O. A. Structural, thermodynamic, and transport properties of aqueous reline and ethaline solutions from molecular dynamics simulations. J. Phys. Chem. B 2019, 123, 11014–11025. 10.1021/acs.jpcb.9b09729. 31794220 PMC6935864

[ref97] Dawass N.; Langeveld J.; Ramdin M.; Pérez-Gallent E.; Villanueva A. A.; Giling E. J.; Langerak J.; Van Den Broeke L. J.; Vlugt T. J. H.; Moultos O. A. Solubilities and Transport Properties of CO2, Oxalic Acid, and Formic Acid in Mixed Solvents Composed of Deep Eutectic Solvents, Methanol, and Propylene Carbonate. J. Phys. Chem. B 2022, 126, 3572–3584. 10.1021/acs.jpcb.2c01425. 35507866 PMC9125562

[ref98] Lyubartsev A. P.; Laaksonen A. Concentration effects in aqueous NaCl solutions. A molecular dynamics simulation. J. Phys. Chem. 1996, 100, 16410–16418. 10.1021/jp961317h.

[ref99] Allen M. P.; Tildesley D. J. Computer Simulation of Liquids; Oxford University Press: Oxford, 1987.

[ref100] Kubisiak P.; Eilmes A. Estimates of Electrical Conductivity from Molecular Dynamics Simulations: How to Invest the Computational Effort. J. Phys. Chem. B 2020, 124, 9680–9689. 10.1021/acs.jpcb.0c07704. 33063509 PMC7604855

[ref101] Loche P.; Steinbrunner P.; Friedowitz S.; Netz R. R.; Bonthuis D. J. Transferable ion force fields in water from a simultaneous optimization of ion solvation and ion–ion interaction. J. Phys. Chem. B 2021, 125, 8581–8587. 10.1021/acs.jpcb.1c05303. 34292738 PMC8389903

[ref102] Hu Z.; Jiang J. Assessment of biomolecular force fields for molecular dynamics simulations in a protein crystal. J. Comput. chem. 2009, 31, 371–380. 10.1002/jcc.21330. 19479737

[ref103] Yllö A.; Zhang C. Experimental and molecular dynamics study of the ionic conductivity in aqueous LiCl electrolytes. Chem. Phys. Lett. 2019, 729, 6–10. 10.1016/j.cplett.2019.05.004.

[ref104] Zhang Y.; Maginn E. J. Direct correlation between ionic liquid transport properties and ion pair lifetimes: a molecular dynamics study. journal of physical chemistry letters 2015, 6, 700–705. 10.1021/acs.jpclett.5b00003. 26262489

[ref105] Krienke H.; Opalka D. Hydration of molecular ions: A molecular dynamics study with a SPC/E water model. J. Phys. Chem. C 2007, 111, 15935–15941. 10.1021/jp073721u.

[ref106] Prasad S.; Chakravarty C.; Kashyap H. K. Concentration-dependent structure and dynamics of aqueous LiCl solutions: a molecular dynamics study. J. Mol. Liq. 2017, 225, 240–250. 10.1016/j.molliq.2016.11.042.

[ref107] Kowsari M.; Alavi S.; Ashrafizaadeh M.; Najafi B. Molecular dynamics simulation of imidazolium-based ionic liquids. II. Transport coefficients. J. Chem. Phys. 2009, 130, 014703 10.1063/1.3042279. 19140627

[ref108] Kowsari M.; Alavi S.; Najafi B.; Gholizadeh K.; Dehghanpisheh E.; Ranjbar F. Molecular dynamics simulations of the structure and transport properties of tetra-butylphosphonium amino acid ionic liquids. Phys. Chem. Chem. Phys. 2011, 13, 8826–8837. 10.1039/c0cp02581j. 21455505

[ref109] Mondal A.; Balasubramanian S. A molecular dynamics study of collective transport properties of imidazolium-based room-temperature ionic liquids. Journal of Chemical & Engineering Data 2014, 59, 3061–3068. 10.1021/je500132u.

[ref110] Rey-Castro C.; Vega L. F. Transport properties of the ionic liquid 1-ethyl-3-methylimidazolium chloride from equilibrium molecular dynamics simulation. The effect of temperature. J. Phys. Chem. B 2006, 110, 14426–14435. 10.1021/jp062885s. 16854152

[ref111] Sala J.; Guardia E.; Marti J. Effects of concentration on structure, dielectric, and dynamic properties of aqueous NaCl solutions using a polarizable model. J. Chem. Phys. 2010, 132, 214505 10.1063/1.3429253. 20528029

[ref112] Shao Y.; Shigenobu K.; Watanabe M.; Zhang C. Role of viscosity in deviations from the Nernst-Einstein relation. J. Phys. Chem. B 2020, 124, 4774–4780. 10.1021/acs.jpcb.0c02544. 32412758 PMC7497660

[ref113] Zhang W.; Chen X.; Wang Y.; Wu L.; Hu Y. Experimental and modeling of conductivity for electrolyte solution systems. ACS omega 2020, 5, 22465–22474. 10.1021/acsomega.0c03013. 32923805 PMC7482292

[ref114] Moura-Neto M. H.; Monteiro M. F.; Ferreira F. A.; Silva D. J.; Figueiredo C. S.; Ciambelli J. R.; Pereira L. S.; do Nascimento J. F.; Chiavone-Filho O. Density and Electrical Conductivity for Aqueous Mixtures of Monoethylene Glycol and Sodium Chloride: Experimental Data and Data-Driven Modeling for Composition Determination. Journal of Chemical & Engineering Data 2021, 66, 1914–1928. 10.1021/acs.jced.0c00962.

[ref115] Grasselli F.; Baroni S. Topological quantization and gauge invariance of charge transport in liquid insulators. Nat. Phys. 2019, 15, 967–972. 10.1038/s41567-019-0562-0.

[ref116] Pegolo P.; Grasselli F.; Baroni S. Oxidation States, Thouless’ Pumps, and Nontrivial Ionic Transport in Nonstoichiometric Electrolytes. Physical Review X 2020, 10, 041031 10.1103/PhysRevX.10.041031.

[ref117] Vega C. Water one molecule, two surfaces, one mistake. Mol. Phys. 2015, 113, 1145 10.1080/00268976.2015.1005191.

[ref118] Predota M.; Biriukov D. Electronic continuum correction without scaled charges. J. Mol. Liq. 2020, 314, 113571 10.1016/j.molliq.2020.113571.

[ref119] Jorge M.; Lue L. The dielectric constant: Reconciling simulation and experiment. J. Chem. Phys. 2019, 150, 084108 10.1063/1.5080927. 30823777

[ref120] Einstein A. Über die von der molekularkinetischen Theorie der Wärme geforderte Bewegung von in ruhenden Flüssigkeiten suspendierten Teilchen. Annalen der physik 1905, 322, 549 10.1002/andp.19053220806.

[ref121] Yeh I. C.; Hummer G. System-Size Dependence of Diffusion Coefficients and Viscosities from Molecular Dynamics Simulations with Periodic Boundary Conditions. J. Phys. Chem. B 2004, 108, 15873 10.1021/jp0477147.

[ref122] Celebi A. T.; Jamali S. H.; Bardow A.; Vlugt T. J. H.; Moultos O. A. Finite-size effects of diffusion coefficients computed from molecular dynamics: a review of what we have learned so far. Mol. Simul. 2021, 47, 831–845. 10.1080/08927022.2020.1810685.

[ref123] Moultos O. A.; Zhang Y.; Tsimpanogiannis I. N.; Economou I. G.; Maginn E. J. System-size corrections for self-diffusion coefficients calculated from molecular dynamics simulations: The case of CO2, n-alkanes, and poly (ethylene glycol) dimethyl ethers. J. Chem. Phys. 2016, 145, 074109 10.1063/1.4960776. 27544089

[ref124] Jamali S. H.; Hartkamp R.; Bardas C.; Sohl J.; Vlugt T. J. H.; Moultos O. A. Shear viscosity computed from the finite-size effects of self-diffusivity in equilibrium molecular dynamics. J. Chem. Theory Comput. 2018, 14, 5959–5968. 10.1021/acs.jctc.8b00625. 30296092 PMC6236468

[ref125] van der Spoel D.; Lindahl E.; Hess B.; Groenhof G.; Mark A. E.; Berendsen H. J. C. Gromacs: Fast, flexible and free. J. Comput. Chem. 2005, 26, 1701 10.1002/jcc.20291. 16211538

[ref126] Hess B.; Kutzner C.; van der Spoel D.; Lindahl E. GROMACS 4: Algorithms for Highly Efficient, Load-Balanced, and Scalable Molecular Simulation. J. Chem. Theory Comput. 2008, 4, 435–447. 10.1021/ct700301q. 26620784

[ref127] Beeman D. Some multistep methods for use in molecular dynamics calculations. J. Comput. Phys. 1976, 20, 130–139. 10.1016/0021-9991(76)90059-0.

[ref128] Nosé S. A molecular dynamics method for simulations in the canonical ensemble. Mol. Phys. 1984, 52, 255–268. 10.1080/00268978400101201.

[ref129] Hoover W. G. Canonical dynamics: equilibrium phase-space distributions 1985, 31, 1695–1697. 10.1103/PhysRevA.31.1695. 9895674

[ref130] Parrinello M.; Rahman A. Polymorphic Transitions in Single Crystals: A New Molecular Dynamics Method 1981, 52, 7182–7190. 10.1063/1.328693.

[ref131] Essmann U.; Perera L.; Berkowitz M. L.; Darden T.; Lee H.; Pedersen L. G. A smooth particle mesh Ewald method. J. Chem. Phys. 1995, 103, 8577–8593. 10.1063/1.470117.

[ref132] Hess B.; Bekker H.; Berendsen H. J. C.; Fraaije J. G. E. M. LINCS: A linear constraint solver for molecular simulations. J. Comput. Chem. 1997, 18, 1463 10.1002/(SICI)1096-987X(199709)18:12<1463::AID-JCC4>3.0.CO;2-H.

[ref133] Hess B. P-LINCS: A Parallel Linear Constraint Solver for molecular simulation. J. Chem. Theory Comput. 2008, 4, 116–122. 10.1021/ct700200b. 26619985

[ref134] Delft High Performance Computing Centre (DHPC), DelftBlue Supercomputer (Phase 1). https://www.tudelft.nl/dhpc/ark:/44463/DelftBluePhase1, 2022.

[ref135] Plimpton S. Fast Parallel Algorithms for Short-Range Molecular Dynamics. J. Comput. Phys. 1995, 117, 1–19. 10.1006/jcph.1995.1039.

[ref136] Ryckaert J. P.; Ciccotti G.; Berendsen H. J. Numerical integration of the cartesian equations of motion of a system with constraints: molecular dynamics of n-alkanes. J. Comput. Phys. 1977, 23, 327–341. 10.1016/0021-9991(77)90098-5.

[ref137] Frenkel D.; Smit B. Understanding molecular simulation: from algorithms to applications, 2nd ed.; Elsevier: San Diego, 2002.

[ref138] Hockney R.; Eastwood J. Computer Simulation Using Particles, 1st ed.; CRC Press: New York, 1988.

[ref139] Plimpton S. LAMMPS Documentation (15 Sep 2022 version). 2015; https://docs.lammps.org/Manual.html (accessed 26/10/2022).

[ref140] Martinez L.; Andrade R.; Birgin E. G.; Martínez J. M. PACKMOL: A package for building initial configurations for molecular dynamics simulations. J. Comput. Chem. 2009, 30, 2157–2164. 10.1002/jcc.21224. 19229944

[ref141] Jamali S. H.; Wolff L.; Becker T. M.; de Groen M.; Ramdin M.; Hartkamp R.; Bardow A.; Vlugt T. J. H.; Moultos O. A. OCTP: ATool for On-the-Fly Calculation of Transport Properties of Fluids with the order-*n* Algorithm in LAMMPS. J. Chem. Inf. Model. 2019, 59, 1290–1294. 10.1021/acs.jcim.8b00939. 30742429

[ref142] Dubbeldam D.; Ford D. C.; Ellis D. E.; Snurr R. Q. A new perspective on the order-*n* algorithm for computing correlation functions. Mol. Simul. 2009, 35, 1084–1097. 10.1080/08927020902818039.

[ref143] Chambers J.; Stokes J. M.; Stokes R. Conductances of concentrated aqueous sodium and potassium chloride solutions at 25. J. Phys. Chem. 1956, 60, 985–986. 10.1021/j150541a040.

[ref144] Gullbrekken Ø.; Røe I. T.; Selbach S. M.; Schnell S. K. Charge Transport in Water–NaCl Electrolytes with Molecular Dynamics Simulations. J. Phys. Chem. B 2023, 127, 2729–2738. 10.1021/acs.jpcb.2c08047. 36921121 PMC10068734

[ref145] Jorge M.; Barrera M. C.; Milne A. W.; Ringrose C.; Cole D. J. What is the Optimal Dipole Moment for Nonpolarizable Models of Liquids?. J. Chem. Theory Comput. 2023, 19, 1790–1804. 10.1021/acs.jctc.2c01123. 36827585 PMC10061682

[ref146] Liu H.; Wang Y.; Bowman J. M. Transferable ab initio dipole moment for water: three applications to bulk water. J. Phys. Chem. B 2016, 120, 1735–1742. 10.1021/acs.jpcb.5b09213. 26436449

[ref147] Liu H.; Wang Y.; Bowman J. M. Quantum calculations of the IR spectrum of liquid water using ab initio and model potential and dipole moment surfaces and comparison with experiment. J. Chem. Phys. 2015, 142, 194502 10.1063/1.4921045. 26001464

[ref148] Laliberté M. Model for calculating the viscosity of aqueous solutions. J. Chem. Eng. Data 2007, 52, 321–335. 10.1021/je0604075.

[ref149] Müller K.; Hertz H. A parameter as an indicator for water- water association in solutions of strong electrolytes. J. Phys. Chem. 1996, 100, 1256–1265. 10.1021/jp951303w.

[ref150] Jamali S. H.; Wolff L.; Becker T. M.; Bardow A.; Vlugt T. J. H.; Moultos O. A. Finite-size effects of binary mutual diffusion coefficients from molecular dynamics. J. Chem. Theory Comput. 2018, 14, 2667–2677. 10.1021/acs.jctc.8b00170. 29664633 PMC5943679

[ref151] Jamali S. H.; Bardow A.; Vlugt T. J. H.; Moultos O. A. Generalized form for finite-size corrections in mutual diffusion coefficients of multicomponent mixtures obtained from equilibrium molecular dynamics simulation. J. Chem. Theory Comput. 2020, 16, 3799–3806. 10.1021/acs.jctc.0c00268. 32338889 PMC7288667

[ref152] French M.; Hamel S.; Redmer R. Dynamical screening and ionic conductivity in water from ab initio simulations. Physical review letters 2011, 107, 185901 10.1103/PhysRevLett.107.185901. 22107646

[ref153] Hansen J.-P.; McDonald I. R. Theory of simple liquids: with applications to soft matter; Academic Press, 2013.

[ref154] Baştuğ T.; Kuyucak S. Temperature dependence of the transport coefficients of ions from molecular dynamics simulations. Chemical physics letters 2005, 408, 84–88. 10.1016/j.cplett.2005.04.012.

[ref155] Bader R.; A quantum theory; Clarendon: Oxford, UK, 1990.

[ref156] Sanville E.; Kenny S. D.; Smith R.; Henkelman G. Improved grid-based algorithm for Bader charge allocation. Journal of computational chemistry 2007, 28, 899–908. 10.1002/jcc.20575. 17238168

[ref157] Krishnamoorthy A.; Nomura K.-i.; Baradwaj N.; Shimamura K.; Rajak P.; Mishra A.; Fukushima S.; Shimojo F.; Kalia R.; Nakano A.; et al. Dielectric constant of liquid water determined with neural network quantum molecular dynamics. Phys. Rev. Lett. 2021, 126, 216403 10.1103/PhysRevLett.126.216403. 34114857

[ref158] Piaggi P. M.; Weis J.; Panagiotopoulos A. Z.; Debenedetti P. G.; Car R. Homogeneous ice nucleation in an ab initio machine-learning model of water. Proc. Natl. Acad. Sci. U. S. A. 2022, 119, e2207294119 10.1073/pnas.2207294119. 35939708 PMC9388152

