# Supplementary material for: Computation of Electrical Conductivities of Aqueous Electrolyte Solutions: Two Surfaces, One Property
Source: J Chem Theory Comput. 2023 Jul 28;19(16):5380–93. doi: 10.1021/acs.jctc.3c00562 (PMC10448725; doi:10.1021/acs.jctc.3c00562)
Supplement: Supplementary file 1 — ct3c00562_si_001.pdf [file ct3c00562_si_001.pdf]

Supporting Information
Computation of Electrical Conductivities of Aqueous Electrolyte Solutions: Two Surfaces, One Property

Samuel Blazquez[1], Jose L. F. Abascal[1], Jelle Lagerweij[2], Parsa Habibi[2,3], Poulumi Dey[3], Thijs J. H. Vlugt[2], Othonas A. Moultos[2] and Carlos Vega*[1]

[1]*Dpto. Química Física I, Fac. Ciencias Químicas, Universidad Complutense de Madrid, 28040 Madrid, Spain*

[2]*Engineering Thermodynamics, Process and Energy Department, Faculty of Mechanical, Maritime and Materials Engineering, Delft University of Technology, Leeghwaterstraat 39, 2628CB, Delft, The Netherlands*

[3]*Department of Materials Science and Engineering, Faculty of Mechanical, Maritime and Materials Engineering, Delft University of Technology, Mekelweg 2, 2628CD, Delft, The Netherlands*

***Corresponding author: cvega@quim.ucm.es**

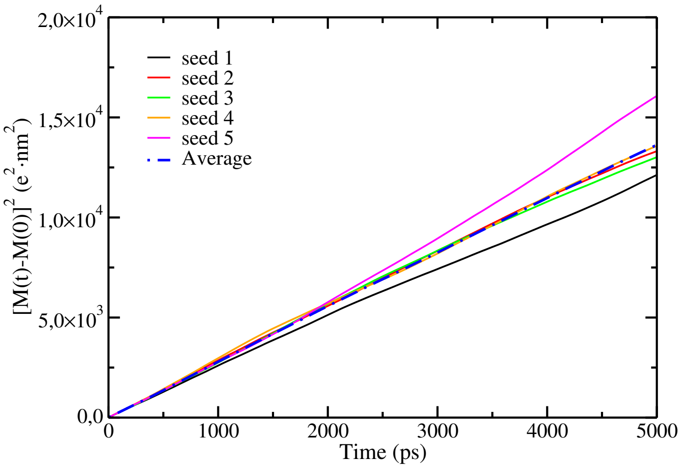

FIG. S1. Mean square dipole displacement in function of time for different seeds of an aqueous NaCl 4 m solution at 298.15 K and 1 bar by using the Madrid-Transport force field. The blue dashed line is the average of the five initial seeds.

TABLE S1. Results for the self-diffusion coefficients of Na^+ and Cl^+ ($\cdot 10^5$ and in cm^2/s) and electrical conductivities calculated with the Nernst-Einstein approximation (σ_{NE+YH}) and by using the Einstein-Helfand equation (σ_{EH}) in $S{\cdot}m^{-1}$ obtained with the different models studied in this work for aqueous solutions of NaCl at $T = 298.15$ K, $p = 1$ bar and at different concentrations below the experimental solubility. Experimental results for electrical conductivities have been taken from Ref.[1]

Molality	JC-SPC/E				SD-SPC/E				Madrid-2019				Experimental
mol/kg	D_{Na}	D_{Cl}	σ_{NE+YH}	σ_{EH}	D_{Na}	D_{Cl}	σ_{NE+YH}	σ_{EH}	D_{Na}	D_{Cl}	σ_{NE+YH}	σ_{EH}	σ_{Expt}
1	1.07	1.38	9.06	6.09	1.27	1.49	10.13	7.12	1.11	1.38	9.15	6.52	8.48
2	0.89	1.14	14.76	9.60	1.11	1.25	16.91	10.72	0.97	1.21	15.72	10.67	14.49
4	0.59	0.74	18.61	12.05	0.82	0.91	23.54	13.35	0.72	0.90	22.43	15.64	22.04
6	0.39	0.46	17.08	11.10	0.62	0.65	24.57	15.00	0.54	0.66	23.91	15.90	25.03

TABLE S2. Computed electrical conductivities (σ in units of S·m^{-1}) of aqueous NaCl solutions from the EH relations for different molalities (m in units of mol$_{\text{salt}}$ kg$_{\text{water}}$$^{-1}$). All simulations are performed at 1 bar and 298.15 K using the Madrid-Transport model. The number of water molecules (n_{W}) and NaCl molecules (n_{s}), the corresponding densities (ρ in units of kg m^{-3}) and viscosities (η in units of mPa·s) are shown for all molalities. Additional electrical conductivities computed using the Nernst-Einstein with ($\sigma_{\text{NE+YH}}$ in units of S·m^{-1}) and without (σ_{NE} in units of S·m^{-1}) Yeh-Hummer finite-size corrections[2–4] are reported as well. We show the results obtained by fitting the mean square dipole displacement between 50-1000 ps (Madrid 1 ns) and between 50-2000 ps (Madrid 2 ns).

	m	n_{w}	n_{s}	ρ	η	σ	σ_{NE}	$\sigma_{\text{NE+YH}}$
Expt.	0	-	-	997.043	0.89	0	-	-
Madrid	0	4440	0	997.3(3)	0.85(5)	0	0	0
Expt	1	-	-	1036.21	0.97	8.48	-	-
Madrid 1 ns	1	4440	80	1035.2(5)	0.97(7)	7.8(1)	9.7(2)	10.6(2)
Madrid 2 ns	1	4440	80	1035.2(5)	0.97(7)	8.0(4)	9.7(2)	10.6(2)
Expt.	2	-	-	1072.27	1.08	14.49	-	-
Madrid 1 ns	2	4440	160	1070.3(5)	1.12(7)	14.0(1)	17.3(1)	18.9(1)
Madrid 2 ns	2	4440	160	1070.3(5)	1.12(7)	14.3(4)	17.3(1)	18.9(1)
Expt.	4	-	-	1136.91	1.35	22.04	-	-
Madrid 1 ns	4	4440	320	1135.4(5)	1.44(10)	20.1(4)	27.3(1)	29.6(1)
Madrid 2 ns	4	4440	320	1135.4(5)	1.44(10)	20.2(5)	27.3(1)	29.6(1)
Expt.	6	-	-	1192.88	1.75	25.03	-	-
Madrid 1 ns	6	4440	480	1194.5(5)	1.79(10)	22.2(8)	32.6(01)	35.2(01)
Madrid 2 ns	6	4440	480	1194.5(5)	1.79(10)	21.9(9)	32.6(01)	35.2(01)

TABLE S3. Computed electrical conductivities (σ in units of S·m^{-1}) of aqueous KCl solutions from the EH relations for different molalities (m in units of mol$_{\text{salt}}$ kg$_{\text{water}}$$^{-1}$). All simulations are performed at 1 bar and 298.15 K using the Madrid-Transport model. The number of water molecules (n_{W}) and KCl molecules (n_{s}), the corresponding densities (ρ in units of kg m^{-3}) and viscosities (η in units of mPa·s) are shown for all molalities. Additional electrical conductivities computed using the Nernst-Einstein with ($\sigma_{\text{NE+YH}}$ in units of S·m^{-1}) and without (σ_{NE} in units of S·m^{-1}) Yeh-Hummer finite-size corrections[2–4] are reported as well. We show the results obtained by fitting the mean square dipole displacement between 50-1000 ps (Madrid 1 ns) and between 50-2000 ps (Madrid 2 ns).

	m	n_{w}	n_{s}	ρ	η	σ	σ_{NE}	$\sigma_{\text{NE+YH}}$
Expt.	0	-	-	997.043	0.89	0	-	-
Madrid	0	4440	0	997.3(3)	0.85(5)	0	0	0
Expt.	2	-	-	1081.5	0.90	19.98	-	-
Madrid 1 ns	2	4440	160	1081.1(5)	0.95(5)	20.4(9)	24.0(1)	25.8(2)
Madrid 2 ns	2	4440	160	1081.1(5)	0.95(5)	19.5(9)	24.0(1)	25.8(2)
Expt.	4	-	-	1152.2	0.94	34.15	-	-
Madrid 1 ns	4	4440	320	1152.3(5)	1.03(7)	32.5(6)	40.5(1)	43.1(1)
Madrid 2 ns	4	4440	320	1152.3(5)	1.03(7)	32.9(6)	40.5(1)	43.1(1)

TABLE S4. Contributions to the electrical conductivities computed by the individual Onsager coefficients (σ in units of S·m^{-1}) of aqueous NaCl solutions from the EH relations for different molalities (m in units of mol$_{salt}$ kg$_{water}$$^{-1}$) and system sizes. All simulations were performed at 1 bar and 298.15 K, using the Madrid-Transport model. The number of water molecules (n_W) and NaCl molecules (n_s) are shown for all molalities. Numbers in parentheses are the uncertainty in the last two digits of the results.

	m	n_w	n_s	σ_{++}	σ_{+-}	σ_{-+}	σ_{--}
Delft	1	1000	18	3.97(18)	-0.068(05)	-0.068(05)	4.91(12)
Delft	1	555	10	3.91(11)	-0.03(01)	-0.03(01)	4.85(19)
Delft	2	1000	36	6.52(15)	0.00(02)	0.00(02)	7.76(25)
Delft	2	555	20	6.06(26)	-0.05(05)	-0.05(05)	7.85(49)
Delft	4	1000	72	8.66(18)	0.17(17)	0.17(17)	11.40(27)
Delft	4	555	40	9.08(27)	0.50(13)	0.50(13)	11.54(36)
Delft	6	1000	108	9.60(29)	0.79(18)	0.79(18)	12.63(25)
Delft	6	555	60	9.82(27)	0.68(08)	0.68(08)	12.61(48)

TABLE S5. Contributions to the electrical conductivities computed by the individual Onsager coefficients (σ in units of S·m^{-1}) of aqueous NaCl solutions from the EH relations for different molalities (m in units of mol$_{salt}$ kg$_{water}$$^{-1}$) and system sizes. All simulations were performed at 1 bar and 298.15 K, using the Madrid-Transport model. The number of water molecules (n_W) and KCl molecules (n_s) are shown for all molalities. Numbers in parentheses are the uncertainty in the last two digits of the results.

	m	n_w	n_s	σ_{++}	σ_{+-}	σ_{-+}	σ_{--}
Delft	2	1000	36	11.18(07)	0.27(02)	0.27(02)	9.10(24)
Delft	2	555	20	11.27(46)	0.36(05)	0.36(05)	8.85(02)
Delft	4	1000	72	16.92(61)	1.29(14)	1.29(14)	13.39(63)
Delft	4	555	40	15.93(04)	1.23(17)	1.23(17)	14.02(66)

[1] Chambers, J.; Stokes, J. M.; Stokes, R. Conductances of concentrated aqueous sodium and potassium chloride solutions at 25. *The Journal of Physical Chemistry* **1956**, *60*, 985–986.

[2] Yeh, I. C.; Hummer, G. System-Size Dependence of Diffusion Coefficients and Viscosities from Molecular Dynamics Simulations with Periodic Boundary Conditions. *J. Phys. Chem. B* **2004**, *108*, 15873.

[3] Celebi, A. T.; Jamali, S. H.; Bardow, A.; Vlugt, T. J. H.; Moultos, O. A. Finite-size effects of diffusion coefficients computed from molecular dynamics: a review of what we have learned so far. *Molecular Simulation* **2021**, *47*, 831–845.

[4] Jamali, S. H.; Hartkamp, R.; Bardas, C.; Sohl, J.; Vlugt, T. J. H.; Moultos, O. A. Shear viscosity computed from the finite-size effects of self-diffusivity in equilibrium molecular dynamics. *Journal of Chemical Theory and Computation* **2018**, *14*, 5959–5968.
